# Decoding the Tumor Microenvironment: Exosome-Mediated Macrophage Polarization and Therapeutic Frontiers

**DOI:** 10.7150/ijbs.114222

**Published:** 2025-06-20

**Authors:** Yilin Li, Jiaqi You, Zifang Zou, Guanghao Sun, Yuqing Shi, Yanbin Sun, Shun Xu, Xin Zhang

**Affiliations:** 1 Department of Thoracic Surgery, The First Hospital of China Medical University, Shenyang 110002, China.; 2 Department of Emergency, The First Hospital of China Medical University, Shenyang 110002, China.; 3 Department of Respiratory Medicine, Shenyang 10th People's Hospital, Shenyang 110096, China.

## Abstract

The tumor microenvironment (TME) is dynamically shaped by interactions between tumor cells, immune cells, and stromal components. Among these, tumor-associated macrophages (TAMs) play dual roles in tumor progression. Exosomes are key mediators of intercellular communication and are crucial for modulating macrophage polarization. This review systematically summarizes the role of HIF-1α as the central regulator of tumor-derived exosomes under hypoxic conditions. Under endoplasmic reticulum stress (ERS), the STAT3 and PI3K/AKT/mTOR pathways activation is mediated by the inactivation of the Hsp90/Hippo pathway, which induces the expression of LncRNA HMMR-AS1 and specific miRNAs (*e.g.,* miR-1246, let-7a, miR-301a-3p, *etc*.). Furthermore, the IRE1/PERK pathway regulates exosome secretion by carrying miR-23a-3p and miR-27a-3p or directly delivering PD-L1 protein, thus activating the PI3K/AKT pathway, inhibiting PTEN, and upregulating PD-L1 expression as well as increasing the M2 polarization of macrophages. This study also summarized the important matrices of exosomes' involvement in the interaction between tumor cells and macrophages in different systemic malignant tumors. Moreover, the bidirectional crosstalk between TAM-derived exosomes and other TME components (*e.g.,* CD8+ T cells, fibroblasts) was also evaluated, which indicated their roles in immune evasion and metastasis. Further, engineering strategies, such as receptor-targeted exosomes and short palindromic repeats interference (CRISPRi)-based transcriptional silencing, were also discussed as emerging tools to enhance exosome specificity and therapeutic efficacy. This study proposes a roadmap for translating engineered exosomes into clinical immunotherapy regimens by integrating recent advances in spatial omics and artificial intelligence, and also addresses challenges in exosome isolation, stability, and biosafety.

## 1. Introduction

Macrophage polarization is a common immunological cellular biological process, where macrophages can manifest distinct functionalities and immune response characteristics under diverse microenvironmental conditions. Macrophage polarization is crucial for various physiological and pathological processes such as inflammation, infection, tissue repair, and tumors (1, 2). Macrophage polarization is characterized by its classification into two polar states: M1 type and M2 type, based on the signals and microenvironments (2, 3), which indicate disparate functions and immune responses. The role of M1-type macrophages in cancer is intricate and is influenced by the tumor microenvironment (TME). Although they might suppress tumor growth in the early phases, M1 macrophages gradually lose their anti-tumor efficacy and even facilitate tumor immune evasion during cancer progression (*4, 5*). M2-type macrophages play a vital role in anti-inflammatory, repair, and immune regulation processes. However, as the tumor progresses, the TME undergoes alterations, and M1-type macrophages are converted into M2-type macrophages (*6, 7*). M2-type macrophages can suppress the immune system's response against tumors. They can significantly increase the expression of programmed death ligand 1 (PD-L1), inhibit T cell activity, and weaken other immune cells' functions, which helps tumor cells evade immune surveillance and attack (*8, 9*). Recent research on the TME has been based on novel technologies, such as Spatial Transcriptomics and Spatial Proteomics, which have provided new perspectives. Recently, a study employed single-cell transcriptomics and spatial proteomics technologies, such as CODEX (a novel high-resolution multiplex imaging technology), and demonstrated that alveolar macrophages develop long-term immune memory following influenza virus infection. Moreover, their polarization state indicated a continuous change gradient rather than being confined to a fixed M1/M2 phenotype (*10*). Furthermore, CODEX technology was employed to identify macrophage subtypes characterized by high VIM expression, which revealed their distinct spatial distribution and functionality within the TME. These findings challenge the applicability of the simplistic M1/M2 classification paradigm (*11*). The single-cell sequencing and spatial transcriptome sequencing analyses of cell-to-cell communication among Selenop-macrophages, cancer-associated fibroblasts (CAFs), CD4+ T cells, and CD8+ T cells indicated a significant increase in the interaction between these cell types in response to immunotherapy for non-small cell lung cancer (NSCLC) (*12*). Therefore, these novel technologies and emerging research perspectives can help address "the limitations of M1-M2 binary classification" and investigate "the interactions among TME cellular components at single-cell resolution" to further elucidate the role of macrophages in the TME.

**Figure 1:**
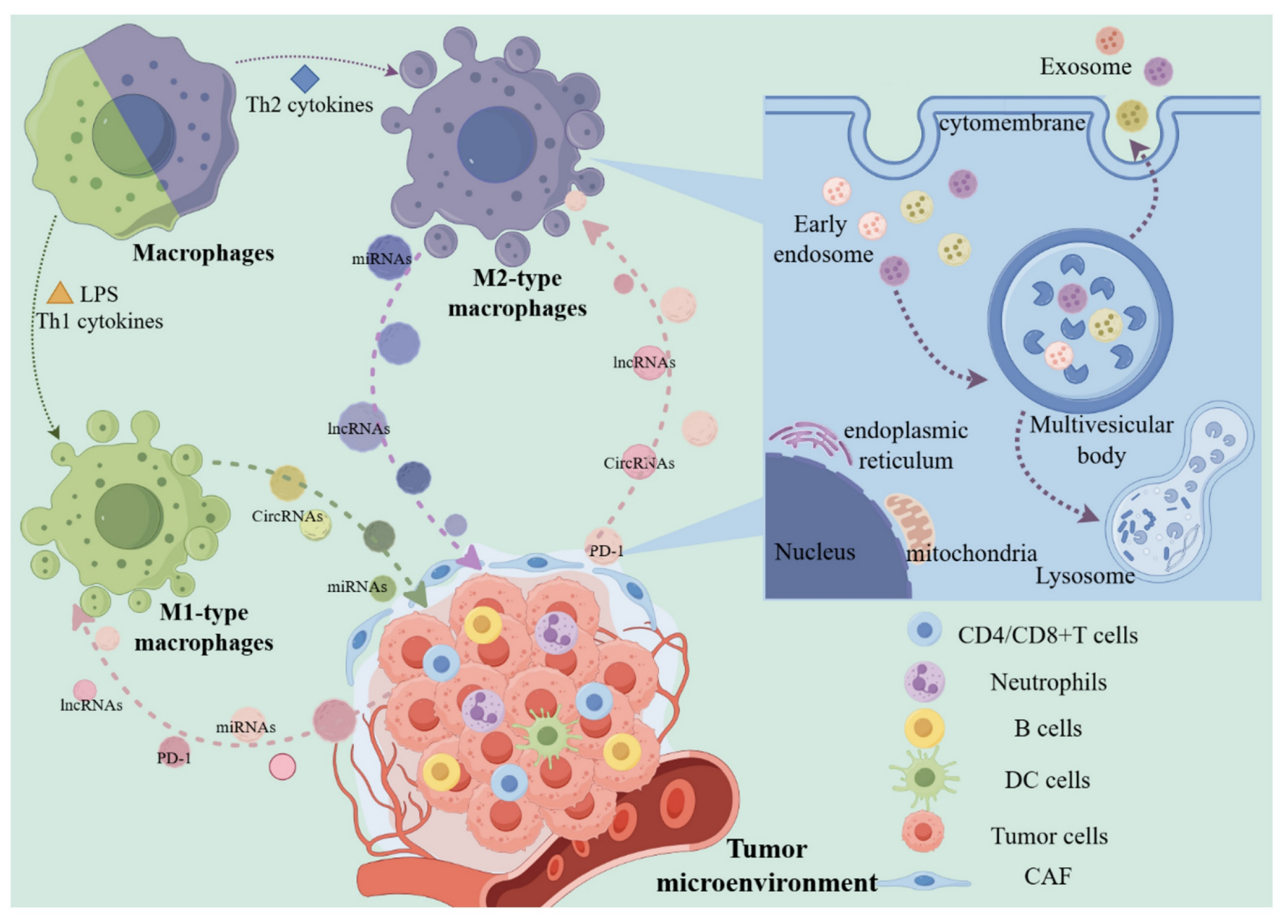
The two polarization types of macrophages and the production process of exosomes. Under the influence of different factors, macrophages are polarized into M1 or M2, and have a promoting or inhibiting effect on tumor cells. In this process, regulatory factors such as exosome-derived proteins and non-coding RNAs play an important role. In macrophages and tumor cells, exosomes encapsulate contents through membrane structures, most of which are decomposed by lysosomes, and some small vesicles are secreted to the extracellular space as mediators to participate in the above-mentioned intercellular interactions. (The Figure was drawn with the help of www.figdraw.com).

Exosomes are small vesicles with a diameter ranging from 30 to 150 nanometers and originate from the endosomal system (*13*). Cells form early endosomes *via* endocytosis, which then transform into late endosomes. In the late endosomes, the membrane buds inwardly to form multiple intraluminal vesicles (ILVs), a portion of which is degraded by lysosomes, while another part is released extracellularly by fusing with the plasma membrane, giving rise to exosomes. The exosomes transport cellular components and facilitate intercellular signaling and material exchange (*14*). Exosome biogenesis pathway differs based on the cargo, cell type, and microenvironment, which directly results in the heterogeneity of exosomes. Several current studies have unveiled the mechanisms influencing exosome biosynthesis, substance loading, and other links. These findings suggest that the different mechanisms are not mutually exclusive, and the same type of cargo can be mediated for exosome sorting through diverse mechanisms (*15*). In TME, the role of exosomes in influencing macrophage polarization is crucial for tumor immunotherapy, potentially reshaping current therapeutic approaches and improving treatment efficacy. Furthermore, the inherent immunomodulatory properties of immune cells-derived exosomes, along with their use in drug delivery and engineering strategies, offer promising avenues for achieving targeted and selective tumor destruction, effective lymph node targeting and regulation, comprehensive immune activation and modulation, as well as ensuring favorable biological safety (*16*) (Figure 1).

## 2. Exosomes Influence the Interaction between Tumor Cells and Macrophages

In addition to common cytokines in the TME, the contents carried by tumor cell-derived exosomes (including proteins, RNA, and lipids) are also significant for the polarization of macrophages. These exosomes cause macrophages to polarize in different directions and therefore affect the tumor's immune microenvironment. This section summarizes the role of tumor-derived exosomes in macrophage polarization and the specific related mechanisms.

### 2.1 Hypoxic Conditions: Exosomes Influence the Interaction between Macrophages and Tumor Cells

The TME characterized by low oxygen levels, or hypoxia, is a distinctive feature of many cancers. This hypoxic condition within tumor tissues, which arises due to rapid cellular proliferation and irregular blood vessel formation, is commonly recognized as a key attribute of solid TMEs. Furthermore, it is also considered a primary factor causing unfavorable prognoses and suboptimal treatment responses in patients (*17*). This hypoxic microenvironment usually activates a series of cellular signaling pathways and molecular mechanisms, influencing the growth, metastasis, and treatment response of tumor cells (*18*). Under hypoxia, the TME components communicate and change with each other, and tumor-associated macrophages (TAMs) preferentially appear in hypoxic areas (*19*). It also regulates the polarization of macrophages. In the communication between various cellular components and macrophages in the hypoxic TME, mediators such as chemokines and exosomes have been proven to play significant regulatory roles (*20*).

Hypoxia-inducible factors (HIFs) are transcription factors induced by hypoxia in cells. HIF1 is the most important member of the HIF family (*21*). It is a crucial modulator in hypoxic tumor cells and exerts complex effects on the transcription of various downstream signaling molecules. Therefore, these changes alter the content of exosomes, influencing the interaction between macrophages and tumor cells. Hypoxic conditions increase HIF-1α expression and its binding to a specific region within the Hsp90 promoter, which enhances Hsp90 expression and elevates the protein levels of Hsp90 in macrophage-derived exosomes (MDEs). The association between Hsp90 and Lats1, an essential component of the Hippo signaling pathway, reduces Lats1 activity, ultimately deactivating the entire Hippo signaling pathway, therefore affecting tumor cell behavior. (*22*). In an oxygen-depleted environment, increased HIF-1α levels can boost the transcription of lncRNA HMMR-AS1 by interacting with its promoter region, thus increasing exosome secretion from hepatocarcinoma cells. The HMMR-AS1 within these exosomes can competitively bind to miR-147a in macrophages, inhibiting ARID3A degradation and facilitating the M2 polarization of macrophages. This process ultimately promotes the progression of hepatocellular carcinoma (HCC) (*23*). M2-type macrophages predominantly utilize mitochondrial oxidative phosphorylation (OXPHOS) as the primary energy source to sustain their immune functions, whereas M1-like macrophages primarily generate ATP *via* glycolysis (*24*). In a pan-cancer investigation, hypoxic tumor exosomes can facilitate the M2-like polarization of infiltrating macrophages. This polarization is driven by the induction of the OXPHOS metabolic profile in macrophages, which is mediated by the transfer of let-7a miRNA to macrophages through the exosome pathway (*25*). In contrast to exosomes derived from normoxic glioma, exosomes derived from hypoxic glioma are most highly enriched in miR-1246. These exosomes can activate the STAT3 signaling pathway and suppress the NF-κB signaling pathway by targeting TERF2IP, which significantly promotes M2 macrophage polarization, facilitating the proliferation, migration, and invasion of glioma both *in vitro* and *in vivo* (*26*). Furthermore, it has been observed that the levels of IL-6 and miR-155-3p in hypoxic exosomes are markedly elevated. IL-6 can enhance autophagy in macrophages by activating STAT3, and miR-155-3p participates in IL-6-induced autophagy by directly targeting CREBRF within macrophages. Both STAT3 activation and the increased autophagy levels in macrophages induce their polarization towards M2, further facilitating tumors' migration, invasion, and in vivo proliferation (*27*). Hypoxic conditions enhance the secretion of exosomes from pancreatic cancer cells. Moreover, the increased levels of miR-301a-3p in exosomes derived from hypoxic environments inhibit PTEN expression in macrophages and upregulate PI3K, p-AKT, and p-mTOR expressions. This leads to M2 polarization of macrophages and enhances the migration, invasion, epithelial-mesenchymal transition, and *in vivo* lung metastasis of pancreatic cancer cells (*28*). Furthermore, exosomes, which originate from epithelial ovarian cancer (OC) cells under hypoxic conditions, are abundant in miR-21-3p, miR-125b-5p, and miR-181d-5p. These exosomes can enhance M2 polarization by activating STAT3 by suppressing SOCS4/5 expression (*29*). In NSCLC, Wang *et al.* discovered that exosomal circPLEKHM1 responds to hypoxia in TME, acting as a messenger in cancer cell-macrophage crosstalk. It promotes the PABPC1-eIF4G interaction to enhance the translation of oncostatin M receptor (OSMR) in macrophages, thereby influencing M2 polarization of macrophages and facilitating NSCLC metastasis. This study reveals how cancer cells in hypoxic TME utilize circRNA-mediated mechanisms to communicate with macrophages and promote tumor progression (*30*). In addition, hypoxia does not facilitate the secretion of exosomes, but it suppresses miR101 levels in lung cancer cells and derived exosomes. The suppression of exosomal miR101 increases CDK8 levels, thus promoting the expression of IL1A, IL6, and M2 polarization in macrophages (*31*). Moreover, the exosomes in the hypoxia culture group facilitate M2 polarization of macrophages by activating the PI3K/AKT signaling pathway. Further investigations disclosed that the level of miR-21 in hypoxia-derived exosomes was significantly increased. Subsequently, exosomal miR-21 promotes M2 polarization of macrophages by targeting IRF1 and suppressing its expression (*32*). However, whether the activation of the PI3K/AKT signaling pathway and the regulation of IRF1 on macrophage polarization are driven by a single factor or result from a joint action remains undetermined. Researchers should investigate the regulatory mechanisms of these two signaling pathways. Furthermore, integrating the cell-cell interactions mediated by exosomes could enhance the rigor of such studies.

### 2.2 Tumor-Derived Exosome-Induced Macrophage Polarization under Endoplasmic Reticulum Stress

When cells experience stress conditions, including hypoxia, nutrient deficiency, or imbalances in calcium ion homeostasis, the function of the endoplasmic reticulum (ER) becomes impaired. This impairment leads to the buildup of unfolded or misfolded proteins within the ER lumen, resulting in a condition known as endoplasmic reticulum stress (ERS). ERS has been shown to influence various pre-cancerous traits and dynamically alter the functions of immune cells. In response to the above stress conditions, the ER's ability to maintain proper protein folding is compromised, accumulating improperly folded proteins, which triggers a state of ER dysfunction, termed ERS. Research has indicated that ERS modulates multiple pre-cancerous characteristics and reshapes the behavior of immune cells in a dynamic manner (*33, 34*). As previously stated, exosomes are derived from the endosomal system and are released as intraluminal vesicles following fusion with the plasma membrane. The molecular mechanisms underlying the formation of secretory endosomes and the subsequent release of exosomes remain poorly understood. Verweij *et al.* identified a subclass of nonproteolytic endosomes as the origin of CD63-positive exosomes at the pre-lysosomal stage. These compartments undergo a sequential activation of Rab7a/ARL8b/Rab27a GTPases, culminating in plasma membrane fusion. During this process, the ER interacts with late endosome membrane contact sites* via* ORP1L, influencing the trafficking and maturation of late endosomes and cooperating with GTPases to regulate these processes (*35*). Therefore, due to the critical role of the ER in the synthesis and release of exosomes, ERS can significantly influence these processes. During ERS conditions, inositol-requiring enzyme 1 (IRE1) downregulates T-cadherin at both the mRNA and protein levels, reducing the production of extracellular vesicles (EVs) (*36*). The formation of multivesicular bodies (MVBs) is crucially involved in cellular processes. Under ERS conditions, MVB formation is modulated by the inhibition of the ERS signal transducers, inositol-requiring enzyme 1 (IRE1) and PKR-like ER kinase (PERK), which is accompanied by an increase in exosome release. Kanemoto *et al.* observed no significant increase in exosome release in IRE1- or PERK-deficient cells, indicating the involvement of IRE1- and PERK-mediated pathways in ERS-dependent exosome release (*37*). ERS also has an important effect on exosomes and their contents. In HCC tissues, the expressions of ERS markers, including glucose-regulated protein 78 (GRP78), activating transcription factor 6 (ATF6), PERK, and IRE1α, are upregulated and have been correlated with an unfavorable prognosis. The exosomes derived from HCC under ERS contain high levels of miR-23a-3p, which can upregulate PD-L1 expression in macrophages by inhibiting PTEN and activating the PI3K/AKT pathway, suppressing the function of T cells. Furthermore, ERS promotes the release of exosomes from HCC cells, further exacerbating PD-L1 expression in macrophages (*38*). Similarly, in breast cancer, exosomal miR-27a-3p upregulates PD-L1 in macrophages to inhibit T cell function; however, this is achieved by miR-27a-3p targeting MAGI2 to inhibit the activation of the PI3K/AKT signaling pathway by PTEN (*39*). Moreover, exosomes derived from ERS in prostate cancer can also activate the PI3K/AKT signaling pathway to promote PD-L1 expression, but the contents carried by the exosomes are unknown (*40*). Exosomes can transport RNA, which in turn, upregulates PD-L1 expression in macrophages. They can also directly convey PD-L1 proteins to boost the PD-L1 levels within these cells. Under ERS conditions, exosomes derived from oral squamous cell carcinoma (OSCC) carry PD-L1 and are delivered directly to macrophages. This process enhances the PD-L1 levels in macrophages, promoting their polarization into the M2 phenotype (*41*). In breast cancer, circular RNA (circ_0001142) is also delivered from tumor cells to macrophages using exosomes as carriers, which regulates autophagy through the miR-361-3p/PIK3CB/PI3K/AKT pathway, inducing M2 polarization and further facilitating tumor proliferation and metastasis (*42*).

**Figure 2:**
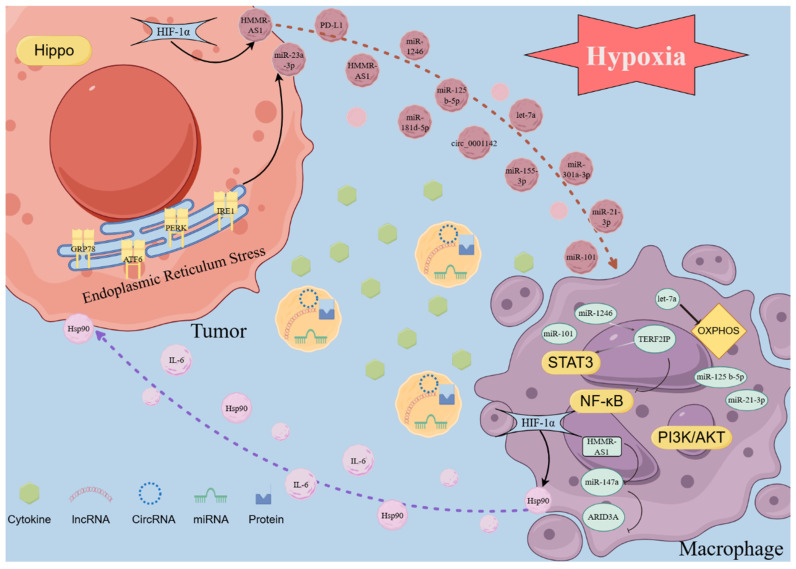
Exosomes in the TME are involved in the interaction between tumor cells and macrophages. In the TME, exosomes produce complex interactions between tumor cells and macrophages under the action of factors such as HIF-1α. In tumor cells, due to ERS, the abnormal expression of corresponding ERS markers affects the contents of exosomes, affects the release of exosomes, and thus affects the cell interaction in TME. The intercellular communication medium of exosomes can indicate the crosstalk between tumor cells and macrophages and the complex biological behaviors in TME in real time. Exosome monitoring can help further understand the intercellular communication of TME to provide a basis for tumor immunotherapy. (The Figure was drawn with the help of www.figdraw.com).

Several researchers have investigated the application of exosome therapy to alleviate ERS in recipient cells to improve the therapeutic efficacy (*43, 44*). In tumors, exosomes can be engineered by incorporating advanced materials to either load them with ERS-relieving drugs or inhibit their regulatory functions, improving the efficacy of immunotherapy. In summary, under ERS conditions, tumor-derived exosomes mainly promote the expression of surface PD-L1 or polarization to M2 by activating the PI3K/AKT pathway in macrophages (Figure 2).

### 2.3 Exosomes from Tumor Cells of Different Systems Promote M1 Polarization of Macrophages

#### 2.3.1 Head and Neck Cancer

Compared with HPV-negative head and neck squamous cell carcinoma (HNSCC), HPV-positive HNSCC can secrete more exosomes, and miR-9 is rich in exosomes derived from HPV+HNSCC. Once miR-9 is picked up by macrophages, it induces M1 macrophage polarization by down-regulating PPARδ and significantly increases the sensitivity of tumor radiotherapy (*45*). Exosomes derived from OSCC significantly promote M1 polarization in macrophages and enhance tumor progression. These exosomes transport THBS1 to stimulate the p38 MAPK, Akt, and SAPK/JNK signaling pathways within macrophages, therefore facilitating M1 polarization. However, when the conditioned medium from these polarized macrophages is used to treat OSCC cells, it increases the tumor cells' migratory capacity (*46*). In conclusion, the M1 polarization of macrophages induced by exosomes has a dual nature, as it can both promote tumor progression and enhance the sensitivity to radiotherapy.

#### 2.3.2 Breast Cancers

In breast cancer, exosomes originating from tumor cells can restrain the tumor progression by polarizing macrophages to the M1 phenotype. Zhang *et al.* discovered that in breast cancer tissues, the tumor suppressor O-type protein tyrosine phosphatase receptor (PTPRO) was correlated with M1 macrophage infiltration. The analysis of breast cancer cell lines with overexpressed PTPRO and inhibited PTPRO-loaded exosomes demonstrated that these exosomes could polarize macrophages to the M1 phenotype by inactivating the STAT signaling pathway with dephosphorylation function, inhibiting the invasion and migration capabilities of breast cancer cells (*47*). Furthermore, Maryam Moradi-Chaleshtori *et al.* established a breast cancer cell line with overexpression of pigment epithelium-derived factor, a tumor suppressor, and revealed that the exosomes originating from it induced M1 polarization of macrophages, augmenting the secretion of M1-specific cytokine IL-1β and reducing the secretion of M2-specific cytokine TGF-β (*48*). They also investigated how miR-130 and miR-33 induced M1 polarization of macrophages related to breast cancer (*49-51*). Moreover, they isolated exosomes from breast cancer cells and utilized electroporation methods to introduce miR-130 and miR-33 mimics into the exosomes individually. The *in vivo* and *in vitro* studies confirmed that the exosomes carrying miR-130 and miR-33 could effectively promote the M1 polarization of macrophages, suppressing tumor migration, invasion, and growth. Overall, exosomes derived from breast cancer can carry proteins or RNA to induce M1 polarization of macrophages, but the specific mechanism remains elusive.

#### 2.3.3 Liver Cancer

Iron oxide nanoparticles (IONs) can modulate the polarization of macrophages towards the M1 phenotype and suppress the proliferation of tumor cells, improving the therapeutic effect of immunotherapy (*52*). Chen and colleagues developed IONs coated with oleic acid through a high-temperature pyrolysis process. Then, PIONs@E6 was produced by incorporating DSPE-PEG 2000 and chlorin e6 into the oleic acid-coated ION solution. The resulting mixture was co-cultured with HCC cells, which yielded exosomes enriched with PIONs@E6. In both cellular and animal models, the exosome-delivered PIONs@E6 effectively synergistically promoted M1 macrophage polarization and suppressed tumor progression (*53*).

### 2.4 Exosomes, Originating from Tumor Cells of Different Systems, Facilitate the M2 Polarization of Macrophages

#### 2.4.1 Head and Neck Cancer

This part will review the aspects of exosomes derived from HNSCC, OSCC, and nasopharyngeal carcinoma in inducing M2 polarization of macrophages.

In HNSCC, it has been proposed that exosomes carrying high levels of PD-L1 can promote the M2 polarization of macrophages and the differentiation of CD4+ T cells to Treg. Furthermore, they also enhance the positive feedback loop of Treg-M2, facilitating the co-generation of Tregs and M2, thus forming an immunosuppressive microenvironment, which promotes tumor growth (*54*). Nils Ludwig *et al.* found that exosomes derived from HNSCC are rich in transforming growth factor β, which can stimulate the chemotaxis of macrophages, but without obvious M1/M2 phenotypic transformation. Instead, it reprograms primary human macrophages into an angiogenic phenotype, characterized by the upregulation of angiogenic factors and functions (*55*). In OSCC tissues, the expression of miR-29a-3p and phosphorylated signal transducer and activator of transcription 6 (p-STAT6) is elevated, whereas the levels of suppressor of cytokine signaling 1 are reduced. At the cellular level, exosomes secreted by OSCC cells transport miR-29a-3p to macrophages, which in turn activate the SOCS1/STAT6 signaling pathway and facilitate M2 polarization of macrophages (*56*). In addition to non-coding RNA, Pang *et al.* discovered that CKLF-like MARVEL transmembrane domain 6 had increased levels in OSCC tissues. The exosomes released by OSCC cells transported CMTM6 to macrophages, promoting their M2-like phenotype polarization by activating the ERK1/2 signaling pathways (*57*).

Cancer stem cells (CSCs), which constitute a small subset of cancer cells, can be distinguished and isolated through the expression of their unique markers. These cells can also secrete exosomes that contribute to tumor development. The exosomes derived from OSCC CSCs can transport lncRNA UCA1, which binds to miR-134 in macrophages, subsequently targeting LAMC2 to modulate the PI3K/AKT signaling pathway within these immune cells, thus promoting their M2 polarization. CSCs can be effectively identified and separated by using their distinctive marker expression. The exosomes they release play a crucial role in facilitating tumor progression (*58*). In nasopharyngeal carcinoma, lncRNA TP73-AS1 is conspicuously highly expressed and is carried into macrophages through exosomes, facilitating the M2 polarization of macrophages and consequently enhancing their migration, invasion, and tube formation (*59*). However, the signaling pathways underlying macrophage polarization warrant further research. PTEN, a well-known tumor suppressor gene, undergoes mutations in various tumors and has been associated with the malignant progression of tumor cells *via* the PI3K/AKT signaling pathway (*60*). Exosomes derived from nasopharyngeal carcinoma can also carry RNF126 to promote the ubiquitination and degradation of PTEN and the activation of the PI3K/AKT pathway, which facilitates the M2 polarization of macrophages (*61*). Altogether, in head and neck tumors, the exosome's content released by tumor cells, such as proteins (PD-L1, CMTM6, RNF126), and RNA (miR-29a-3p, lncRNA UCA1, LncRNA TP73-AS1), can induce M2 polarization of macrophages by activating the SOCS1/STAT6, ERK1/2, and PI3K/AKT pathways, which promotes immunosuppression, proliferation, migration, invasion, and other malignant progressions of the tumor.

#### 2.4.2 Digestive System Tumors

This section focuses on the association of exosomes originating from digestive system tumors, including cholangiocarcinoma (CCA), esophageal, gastric, pancreatic, colorectal, and liver cancers, with the M2 polarization of macrophages. In esophageal squamous cell carcinoma (ESCC) tissues, miR-301a-3p is highly expressed and can enter macrophages *via* exosomes, where it suppresses PTEN and activates the PI3K/AKT signaling pathway to facilitate M2 polarization. Subsequently, M2 macrophages can secrete pro-angiogenic factors such as VEGFA and MMP9, thus promoting tumor angiogenesis (*62*).

In gastric cancer, miR-4435-2HG within exosomes can activate the Jagged1/Notch and JAK1/STAT3 pathways in macrophages to facilitate M2 polarization of macrophages. A study revealed that after co-culturing the M2 polarization macrophages with gastric cancer cells MKN45, the cells manifested a spindle-shaped morphology. Western blotting experiments revealed a marked increase in the expression of N-cadherin and vimentin, suggesting that M2 macrophages induced by exosomes derived from gastric cancer promote the migration of gastric cancer cells by facilitating the EMT process (*63*). Gastric cancer cells can transfer circATP8A1 to macrophages *via* exosomes. CircATP8A1 functions as a molecular sponge, competitively binding to miR-1-3p, alleviating the inhibitory effect of miR-1-3p on STAT6. This process specifically activates the STAT6 signaling pathway and induces tumor-promoting M2 polarization of macrophages. M2 macrophages, in turn, synergistically enhance gastric cancer cells' proliferation, invasion, and immune evasion by secreting immunosuppressive factors (*e.g.,* IL-10, TGF-β) and pro-angiogenic factors (*e.g.,* VEGF). This mechanism highlights the pivotal role of exosome-mediated "tumor cell-macrophage" signaling interactions in TME remodeling (*64*). Ma *et al.* discovered that exosomal ELFN1-AS1 can induce M2 polarization by facilitating the metabolic reprogramming of macrophages. This is primarily achieved by exosomal ELFN1-AS1, which suppresses miR-4644, increasing the expression of PKM, HK2, and GLUT1, as well as glucose uptake and lactate production in macrophages. It has been observed that when the level of exosomal ELFN1-AS1 is reduced, the migratory capacity of macrophages decreases, suggesting that it plays a crucial role in macrophage recruitment (*65*). The metastasis of cancer is a crucial factor for the adverse prognosis of patients.

The formation of pre-metastatic niches (PMN) is one of the most significant steps in cancer metastasis, and exosomes play an important role in PMN formation (*66, 67*). Wang *et al.* discovered that exosomes of mouse gastric cancer cells could enrich in the lung tissue of mice and facilitate the M2 polarization of pulmonary macrophages. This was accomplished by exosomal miR-92a-3p, which inhibits the expression of PTEN and activates the ERK signaling. Therefore, exosomes originating from gastric cancer increase the lung metastasis by promoting the M2 polarization of macrophages (*67*). Xu *et al.* also found that exosomes derived from human gastric cancer cells can accumulate in the liver and be internalized by liver macrophages. Exosomal miR-519a-3p activates the MAPK/ERK pathway by targeting DUSP2, inducing M2-like polarization of macrophages. M2 macrophages accelerate liver metastasis of gastric cancer by inducing angiogenesis and promoting PMN formation in the liver (*66*). In pancreatic cancer, under IL-6 stimulation, the cancer cells release exosomal FGD5-AS1, which is taken up by macrophages, where it can recruit p300 to promote STAT3-K685 acetylation, activate the STAT3/NF-κB signaling pathway, and mediate M2 polarization of macrophages (*68*). In pancreatic cancer, the most common KRAS mutation is G12D (referred to as KRAS G12D). Under oxidative stress, pancreatic ductal adenocarcinoma (PDAC) cells secrete KRAS G12D protein in an autophagy-dependent manner. This protein is then transported to macrophages *via* exosomes. Macrophages internalize exosomal KRAS G12D through the advanced glycation end product-specific receptor, which activates the STAT3 signaling pathway and enhances fatty acid oxidation dependent on this pathway. Subsequently, the macrophages are transformed into M2-like pro-tumor phenotypes (*69*).

Metastasis is common in colorectal cancer (CRC). Lan *et al.* discovered that RPPH1 expression was augmented in CRC samples and was correlated with advanced TNM stage and adverse prognosis. RPPH1 can combine with -III tubulin to prevent its ubiquitination, inducing epithelial-mesenchymal transition to facilitate metastasis. Furthermore, RPPH1 can also be transferred to macrophages *via* exosomes to mediate M2 polarization of macrophages, but the specific mechanism awaits exploration (*70*). Xiong *et al.* used IL-6 stimulation to culture EMT-programmed CRC cells, and then compared the miRNA discrepancies between EMT-derived exosomes and non-EMT-derived exosomes *via* miRNA sequencing. They discovered that the differentially expressed exosomal miR-106b activated the PI3Kγ/Akt/mTOR signaling pathway by directly suppressing programmed cell death 4 (PDCD4), inducing M2 macrophage polarization. Furthermore, the activated M2 macrophages maintained the EMT of CRC cells in a positive feedback manner, enhancing the cells' migration, invasion, and liver and lung metastasis *in vivo* (*71*). The reports suggest that the incidence of liver metastasis of CRC is high. Researchers compared the tissues of CRLM and non-CRLM and discovered that the expression of miR-934 was significantly elevated and was closely associated with liver metastasis. miR-934 can be packaged into the exosomes of CRC cells mediated by hnRNPA2B1 and be internalized by macrophages, where they downregulate PTEN expression and activate the PI3K/AKT signaling pathway to induce M2 polarization of macrophages. Polarized macrophages can secrete CXCL13 to facilitate CRLM. Moreover, CXCL13 can promote miR-934 expression by binding to CXCR5 and activating the positive feedback loop of NFκB/p65/miR-934 (*72*). In addition to the CXCL13/CXCR5 axis, the activation of the CXCL12/CXCR4 axis also plays a crucial role in liver metastasis of CRC (*73*). CXCL12/CXCR4 axis activation upregulates miR-25-3p, miR-130b-3p, and miR-425-5p in exosomes derived from CRC. Consistently, they induce M2 polarization of macrophages by downregulating PTEN expression and activating the PI3K/AKT signaling pathway, enhancing EMT and secreting VEGF to facilitate cancer metastasis (*74*). In the TME of liver cancer, tumor-infiltrating macrophages are one of the most abundant stromal cell types (*75*). Li *et al.* discovered that the lncRNA TUC339 derived from liver cancer exosomes could promote M2 polarization of macrophages. Furthermore, they overexpressed lncRNA TUC339 in macrophages and then performed the GO analysis of differentially expressed genes. The results revealed that genes related to CXCR chemokine receptor binding, cytokine signaling pathways, cell proliferation, immune system defense responses, and regulation of responses to stimuli were significantly enriched. This provided a basis for further research on the specific mechanism of polarization (*76*). Circ00074854 can promote the malignant progression of liver cancer cells. Compared with the control group, exosomes derived from the liver cancer cell line with circ00074854 knockdown can inhibit the M2 polarization of macrophages. The co-culturing of macrophages treated with exosomes from both groups with liver cancer cells showed that the levels of ZEB-1 and vimentin in liver cancer cells were downregulated, while the level of E-cadherin was upregulated. Therefore, it was inferred that exosomes circ00074854 can promote the M2 polarization of macrophages, promoting the migration, invasion, and EMT of liver cancer cells (*77*). The miR-200b-3p of liver cancer cells can enter exosomes mediated by hnRNPA2B1. When M0 macrophages take up the released exosomes, they activate the JAK/STAT signaling pathway by down-regulating ZEB1 and up-regulating the expression of IL-4, facilitating M2 polarization. The M2 macrophages release cytokines with increased expression of PIM1 and VEGFα, which subsequently activate the ERK signaling pathway in tumor cells, promoting the proliferation and metastasis of HCC (*78*). In addition to activating the JAK/STAT signaling pathway, miR-21-5p from HCC-derived exosomes can target RhoB to activate the MAPK/ERK pathway and promote M2 macrophage polarization (*79*). Polarized macrophages not only directly stimulate the malignant progression of tumor cells but also influence the functions of other immune cells within the microenvironment. Zhang *et al.* discovered that miR-146a-5p secreted by HCC cells is delivered to macrophages through exosomes, facilitating their polarization to M2 by activating the STAT3 pathway. M2 macrophages induced by HCC-derived exosomes inhibit the expression of IFN-γ and TNF-α in T cells, while concurrently upregulating the expression of inhibitory receptors such as PD-1 and CTLA-4 in T cells. Furthermore, SALL4 can bind to the promoter of miR-146a-5p and promote its expression, whereas SALL4 silencing suppresses the expression of inhibitory receptors and reverses T cell exhaustion (*80*). CircPOLQ is highly expressed in CRC tissues and correlates with poor patient prognosis. When internalized by macrophages by exosomes, circPOLQ functions as a molecular sponge for miR-379-3p, alleviating its suppression of the IL-10/STAT3 signaling pathway. This process activates STAT3 phosphorylation and induces macrophage polarization toward the pro-tumor M2 phenotype. M2 macrophages promote TME remodeling through the secretion of immunosuppressive factors, such as IL-10, which facilitates the formation of metastatic nodules in CRC. This intercellular interaction mechanism offers a promising therapeutic strategy for targeting the circPOLQ/STAT3 axis or inhibiting exosome-mediated communication (*81*). The digestive system comprises many complex organs. Tumors in these organs can release exosomes encapsulating proteins, miRNA, LncRNA, circRNA, *etc.,* to induce M2 polarization of macrophages and promote tumor progression. The specific activated pathways mainly include the STAT, AKT, and ERK pathways.

#### 2.4.3 Respiratory System Tumors

Lung cancer is clinically categorized into small cell lung cancer (SCLC) and NSCLC. NSCLC is further subdivided into squamous cell carcinoma, adenocarcinoma, large cell carcinoma, and adenosquamous carcinoma. Jessy S. Deshane *et al.* found that exosomes derived from lung tumor cells were uptaken by M0 macrophages in a time-dependent manner and then polarized into M2 macrophages. The comparison between p53-deficient human lung cancer cell line (H358) and the adenocarcinoma human alveolar basal epithelial cell (A549) showed that p53 could regulate exosome generation, and exosome-mediated M2 polarization was independent of the p53 status. Mechanistically, cancer-derived exosomes regulate transcriptional alterations and reprogram macrophage metabolism, promoting their differentiation into the M2 phenotype (*82*). In lung adenocarcinoma (LUAD), exosomal miR-19b-3p secreted by LUAD cells can target macrophage PTPRD, which inhibits PTPRD-mediated STAT3 dephosphorylation in macrophages, resulting in STAT3 activation and M2 polarization. The activated STAT3 in polarized M2 macrophages can transcriptionally induce the LINC00273 expression and transfer it back to LUAD cells in the form of exosomes. In LUAD cells, LINC00273 recruits NEDD4 to facilitate LATS2 ubiquitination and degradation, inactivating the Hippo pathway and activating YAP, which can also induce RBMX transcription. RBMX can encapsulate miR-19b-3p in exosomes derived from LUAD cells. Overall, exosomes serve as exchange media for transferring non-coding RNAs between tumor cells and macrophages, forming a positive feedback loop that promotes tumor progression (*83*). Moreover, the high LINC00963 expression in LUAD facilitates tumor progression *via* two mechanisms. Firstly, the LINC00963/HNRNPA2B1 interaction suppresses ubiquitination-mediated degradation of Zeb1 by degrading Siah1 mRNA, promoting the malignant and metastatic phenotypes. Secondly, LINC00963 can also enter LUAD cells and be released into exosomes, where it induces M2 polarization of macrophages and stimulates macrophage-induced growth of LUAD cells (*84*). In addition to influencing the progression of LUAD, M2-polarized macrophages can also promote resistance to immunotherapy. Ding *et al.* discovered that the level of circZNF451 in exosomes from advanced LUAD patients with resistance to anti-PD-1 drugs was elevated. The circZNF451 transferred to macrophages could elicit M2 polarization of macrophages and suppress the cytotoxicity of CD8+ T cells through TRIM56-mediated ubiquitination of FXR1 and activation of the ELF4-IRF4 pathway. In transgenic mice with high circZNF451 expression in LUAD, the effect of anti-PD-1 treatment increased when ELF4 was conditionally knocked out in macrophages (*85*). Exosomal circSHKBP1 regulates glycolysis *via* the HIF-1 and PKM2 pathways in NSCLC cells, influencing tumor progression. Furthermore, circSHKBP1 secreted by tumor cells through exosomes enhances the expression of PKM2, HK2, and GLUT1 proteins, as well as increases glucose uptake and lactate production in macrophages. This process promotes M2 polarization and macrophage recruitment, affecting NSCLC progression through intercellular communication (*86*). CircATP9A is encapsulated into EVs by the RNA-binding protein hnRNPA2B1 in NSCLC cells. These EVs are subsequently internalized by TAMs, where circATP9A binds to the HuR protein to form a complex that upregulates the expression of the NUCKS1 gene and activates the downstream PI3K/AKT/mTOR signaling pathway. This process induces TAM polarization toward the tumor-promoting M2 phenotype, which then promotes NSCLC progression by secreting immunosuppressive and cancer-promoting factors, establishing a microenvironment conducive to tumor growth and invasion. These findings provide potential therapeutic strategies for targeting the circATP9A/hnRNPA2B1 axis or inhibiting EV-mediated delivery (*87*). A high ratio of M2 macrophages has been associated with metastasis and poor prognosis in SCLC patients. Further, SCLC-derived exosomes facilitate M2 polarization of macrophages* via* the NLRP6/NF-κB signaling pathway. *In vivo* experiments in mice have verified that SCLC-derived exosomes can promote SCLC metastasis (*88*). Altogether, in lung cancer, the influences of LUAD- and SCLC-derived exosomes on the M2 polarization of macrophages have been preliminarily expounded. Exosomes from other types of lung cancer still warrant research.

#### 2.4.4 Urinary and Reproductive System Tumors

Urogenital system tumors are those that arise within the urogenital system. The high incidence of tumors in the urinary system has been observed in the kidneys, bladder, prostate, testicles, *etc.,* and in the reproductive system, which includes the cervix, ovaries, and breasts (*89*). Exosomal lncRNA-ARSR derived from renal cell carcinoma directly interacts with miR-34/miR-449, increases STAT3 expression, promotes M2 polarization of macrophages, and exhibits a phenotype that promotes angiogenesis and tumor growth (*90*). Exosomes AP000439.2 originated from clear cell renal cell carcinoma, directly interact with STAT3 protein and p-STAT3 in macrophages, and activate the NF-κB signaling pathway to facilitate M2 polarization of macrophages. These M2 macrophages are characterized by increased TGF-β and IL-10 levels and can promote the migratory capacity of tumor cells (*91*).

In bladder cancer, exosomal miR-92b-3p and miR-1231-5p derived from the murine bladder cancer cell line MB49 can induce macrophage polarization into an immunosuppressive phenotype by inhibiting PTEN expression and activating the AKT/STAT3/6 pathway, suppressing the proliferation of CD4+T and CD8+T cells, and facilitating tumor growth (*92*). Tyrosine kinase-expressing macrophages that express immunoglobulin and epidermal growth factor homology domain 2 are a subset of TAMs, characterized by their location near newly formed tumor blood vessels and their promotion of angiogenesis (*93*).

Cervical cancer cells with high TIE2 expression reach macrophages as exosomes and induce them to become TEM. Angiopoietin accelerates the formation of TEM and induces macrophage polarization to M2 by activating AKT and PAK1 signaling pathways (*94*). Exosomal miR-423-3p derived from cervical cancer can also inhibit STAT3 phosphorylation by targeting cyclin-dependent kinase 4, which inhibits M2 polarization of macrophages and reduces IL-6 expression, which in turn inhibits tumor progression (*95*).

In OC, miR-1246 promotes tumor progression in multiple ways. Firstly, miR-1246 increases PDGFRβ levels by targeting Cav1, and the high PDGFRβ expression is correlated with a poor disease-free survival rate in OC patients. Secondly, miR-1246 can also target Cav1 to enhance the expression of multidrug resistance protein 1, conferring paclitaxel resistance to OC cells. Finally, miR-1246 can be transferred into M2-type macrophages as exosomes, targeting Cav1 to promote the expression of p-gp and further increase drug resistance (*96*). Exosomes extracted from the blood of OC patients indicated that miR-200b had significantly higher expression than that of healthy volunteers. Exosomal miR-200b can promote M2 polarization and inhibit M1 polarization of macrophages by suppressing KLF6. The culture medium of polarized macrophages promoted the proliferation and invasion of OC cells (*97*).

Breast cancer is the most common malignant tumor that threatens the life and health of women worldwide (*98*). Breast cancer-derived exosomal miR-138-5p downregulates KDM6B expression in macrophages, which results in the polarization of macrophages towards the M2 type and the reduction of KDM6B recruitment to the promoters of M1-related genes. *In vivo* experiments have demonstrated that M2 macrophages polarized by exosomal miR-138-5p can promote lung metastasis of breast cancer (*99*). Furthermore, exosomal miR-222 derived from doxorubicin-resistant MCF-7 breast cancer cells can activate M2 polarization of macrophages by targeting PTEN to promote Akt expression, promoting migration, proliferation, invasion of tumor cells, and the formation of PMN *in vivo*, which stimulates tumor growth (*100*).

#### 2.4.5 Other Systemic Tumors

Exosomes originated from osteosarcoma MG63 cells can induce M2 polarization of macrophages by modulating Tim-3 expression. The differentiated macrophages facilitate the migration, invasion, epithelial-mesenchymal transition, and distant lung metastasis capacity of osteosarcoma cells by secreting IL-10, TGF-β, and VEGF (*101*). Compared with non-metastatic osteosarcoma cell lines (K7 and Dunn), exosomes derived from metastatic cell lines (K7M3 and DLM8) increased TGFB2 expression in macrophages, inducing M2-type macrophages with increased expression of IL10, TGFB2, and CCL22, and reduced phagocytosis, endocytosis, and macrophage-mediated tumor cell killing (*102*). In glioma, miR-3591-3p has been found to act as a tumor suppressive signal. However, when excreted from cells as exosomes, its inhibitory effect on MAPK1 is reduced, which promotes glioma progression by activating the MAPK pathway. Moreover, the released exosomal miR-3591-3p enters macrophages, directly inhibits the expression of GBLB, and then activates the JAK2/PI3K/AKT/mTOR and STAT3 pathways to promote M2 polarization of macrophages. Subsequently, these polarized macrophages secrete IL-10 and TGFβ, thus facilitating the invasion and migration of glioma (*103*). Exosomes derived from diffuse large B-cell lymphoma can carry GP130, the receptor protein of IL-6, enter macrophages, and then activate the STAT3 signaling pathway to promote M2 polarization of macrophages (*104*). In acute myeloid leukemia, a circular RNA, circ001264, is significantly overexpressed. It enters macrophages through the exosome pathway, activates the p38/STAT3 signaling pathway, induces M2 macrophage polarization, and upregulates PD-L1 levels on the surface of macrophages, further inhibiting T cells' immune killing (*105*). The cystine-glutamate exchange in melanoma can enhance glutamate release to balance the oxidative homeostasis within tumor cells and facilitate tumor progression. When the cystine-glutamate transporter (xCT) is inhibited, it disrupts the cellular redox homeostasis, resulting in ROS-induced cell death. However, blocking xCT with SAS-type drugs can lead to the expression of transcription factors IRF4 and EGR1 in melanoma cells and promote PD-L1 levels by binding to its promoter. High PD-L1 levels are secreted in the form of exosomes and then induced on macrophages, where they promote M2 macrophage polarization, and ultimately induce resistance to anti-PD-1/PD-L1 therapy (*106*) (Table 1).

### 2.5 The Role of M1 Macrophage-Derived Exosomes in the Progression of Different Systemic Tumors

The mechanism of action of M1 macrophage exosomes may vary in different types of tumors. For instance, in lung cancer, M1 macrophage exosomes can inhibit tumor growth by suppressing tumor angiogenesis and promoting its apoptosis. In liver cancer, these exosomes can enhance anti-tumor immune responses by regulating immune cells in the TME. Furthermore, M1 macrophage exosomes can inhibit tumor progression by influencing the metabolism and signaling pathways of tumor cells.

In the TME, MMP16 promotes tumor cell invasion and metastasis by degrading matrix components. Yan *et al.* found that miR-150 is highly expressed in exosomes derived from M1 macrophages and can be taken up by tumor cells in large quantities, therefore inhibiting the proliferation of glioblastoma cells by targeting and binding to MMP16 (*107*). Jiang *et al.* studied the functions of M1-derived exosomes and their specific mechanisms in HNSCC. They performed a series of *in vitro* and *in vivo* experiments to study the functional roles of M1-derived exosomes and their key molecule HOTTIP (HOXA transcript at the distal tip, a long non-coding RNA) in HNSCC. The dual-luciferase assay and *in vitro* experiments revealed that HOTTIP was upregulated in M1-derived exosomes. HOTTIP overexpression further enhanced the ability of M1 exosomes to inhibit the proliferation, migration, and invasion of cancer cells, while inducing their apoptosis, indicating that HOTTIP is a key molecule in M1 exosomes. Moreover, the mechanistic studies revealed that HOTTIP in M1 exosomes upregulated the TLR5/NF-κB signaling pathway by competitively binding miR-19a-3p and miR-19b-3p, inhibiting the progression of HNSCC. Furthermore, cancer cells expressing HOTTIP and M1 exosomes reinduced circulating monocytes to express the M1 phenotype, providing new insights into HNSCC (*108*). Wang *et al.* discovered that when mouse breast cancer cells (4T1) were co-cultured with naive macrophages (Mφ) along with M1 macrophage-derived exosomes (M1-EXOs) or M2 macrophage-derived exosomes (M2-EXOs), the M1-EXOs prompted Mφ to adopt a Th1 phenotype, thus promoting the release of pro-inflammatory cytokines and a substantial rise in caspase-3 activity within tumor cells, which indicated enhanced apoptosis. Whereas M2-EXOs produced opposing effects. M1-EXOs created a pro-inflammatory environment and facilitated tumor cell apoptosis *via* a caspase-3-dependent mechanism, exerting anti-tumor properties. M1-EXOs also indicated anti-tumor efficacy *in vivo* in mice bearing tumors (*109*). M1 exosomes can also boost the effectiveness of anti-cancer vaccines. Cheng *et al.* discovered that after subcutaneous administration, M1 exosomes prefer to migrate to lymph nodes, where local macrophages and dendritic cells predominantly internalize them, which triggers the secretion of various Th1 cytokines. Their findings also revealed that M1 exosomes augmented the performance of Trp2 vaccines encapsulated within lipid calcium phosphate nanoparticles, eliciting a more robust antigen-specific cytotoxic T cell response. M1 exosomes outperformed CpG oligonucleotides as immune enhancers in experiments aimed at inhibiting melanoma growth. Overall, this research suggests that exosomes derived from M1-polarized macrophages hold potential as adjuvants for enhancing anti-cancer vaccines (*110*). Kim *et al.* employed an exosome-guided *in situ* direct reprogramming strategy to transform M2-polarized TAMs that support tumor invasion and progression in the TME into M1-type macrophages that attack tumors. Exosomes from M1-type macrophages efficiently promoted the phenotypic transformation of anti-inflammatory M2-like TAMs into pro-inflammatory M1-type macrophages. The reprogrammed M1 macrophages had a protein expression profile similar to classically activated M1 macrophages. Furthermore, it had significantly enhanced phagocytic function and strong cross-presentation ability, improving anti-tumor immunity around the tumor (*111*).

### 2.6 The Role of M2 Macrophage-Derived Exosomes in the Progression of Different Systemic Tumors

Zheng *et al.* found increased accumulation of M2-polarized phenotype TAMs in the gastric cancer microenvironment, a unique immune cell population expressing apolipoprotein E (ApoE). ApoE is a specific and effective protein that mediates intercellular transfer in gastric cancer cells through M2 MDEs, activates the PI3K-Akt signaling pathway, and remodels the cytoskeleton to support and promote the migration of gastric cancer cells (*112*). In esophageal carcinoma, exosomes derived from M2 macrophages can carry high levels of LncRNA AFAP1-AS1 into esophageal carcinoma cells to sponge miR-26a and promote the expression of ATF2, facilitating the migration, invasion, and lung metastasis of EC cells (*113*).

Consistently, the expression of circ0020256 in the supernatant exosomes isolated from THP-1 cells induced into M2 macrophages was significantly increased. The increased circ0020256 levels promoted the expression of the transcription factor E2F3 by interacting with the miRNA target miR-432-5p within CCA cells, therefore inducing the proliferation, migration, and invasion of CCA cells (*114*). In HCC, M2 exosomes carrying miR-660-5p enhance the proliferation, migration, and invasion of HCC cells by down-regulating KLF3 in liver cancer cells. Moreover, M2 MDEsmiR-27a-3p can further advance HCC by targeting TXNIP, improving the CSC properties of HCC (*115, 116*). Lu *et al.* co-cultured HCC cells, human umbilical vein endothelial cells (HUVECs), and M2 macrophages and analyzed the miRNA expression profile of M2 MDEs. They discovered that M2 macrophage-derived miR-23a-3p enhanced the metastasis of HCC by promoting EMT and angiogenesis as well as increasing vascular permeability. They postulated that the infiltration of M2 macrophages was correlated with metastasis and poor prognosis in HCC patients. Moreover, HCC cells co-cultured with M2-derived exosomes secreted increased levels of GM-CSF, VEGF, G-CSF, MCP-1, and IL-4, further recruiting M2 macrophages (*117*). In hepatitis B virus (HBV)-related HCC, Tao *et al.* found that HBeAg secreted by HBV+HCC cells upregulated the expression of lncRNA MAPKAPK5-AS1 (MAAS) in M2 macrophages. M2 MDEs transferred the increased MAAS to HBV+HCC cells, promoting the proliferation of HBV+HCC cells in vitro and in vivo by stabilizing c-Myc protein (*118*). Shen et al. also found that exosomes derived from TAMs in liver cancer tissues can transfer lncMMPA, promoting the growth of HCC tumors in vivo by stimulating the glycolytic pathway. This is achieved through the interaction of lncMMPA with miR-548, targeting ALDH1A3 (*119*). The migration and invasion of CRC cells regulated by M2 macrophages depend on the high expression levels of miR-21-5p and miR-155-5p in M2 MDEs. miR-21-5p and miR-155-5p can target BRG1 and downregulate its expression (*8*). Moreover, after miR-155-5p is transferred to colon cancer cells through M2 MDEs, it targets ZC3H12B and negatively regulates the stability of IL-6 mRNA, increases IL-6 release by tumor cells, and suppresses T-cell immune responses, promoting immune escape in colon cancer (*120*). Yang *et al.* found that M2 macrophages were positively correlated with microvessel density in PDAC tissues. The levels of miR-155-5p and miR-221-5p in M2 MDEs were higher than those in M0 MDEs. Moreover, they could be transferred into mouse aortic endothelial cells and promote angiogenesis of MAECs *in vitro*. Similarly, M2 MDEs were observed to promote the growth of subcutaneous tumors and increase vascular density in mice. This discovery may provide a strategy for the clinical treatment of PDAC by targeting M2 MDEs (*121*). Some studies revealed that miR-501-3p in M2 MDEs inhibited the tumor suppressor gene TGFBR3 by activating the TGF-β signaling pathway, promoting PDAC development (*122*).

Table 2 summarizes the details of how M2 MDEs affect other tumors.

## 3. Exosomes Participate in the Interactions between Other Components of the Tumor Microenvironment and Macrophages

**Figure 3:**
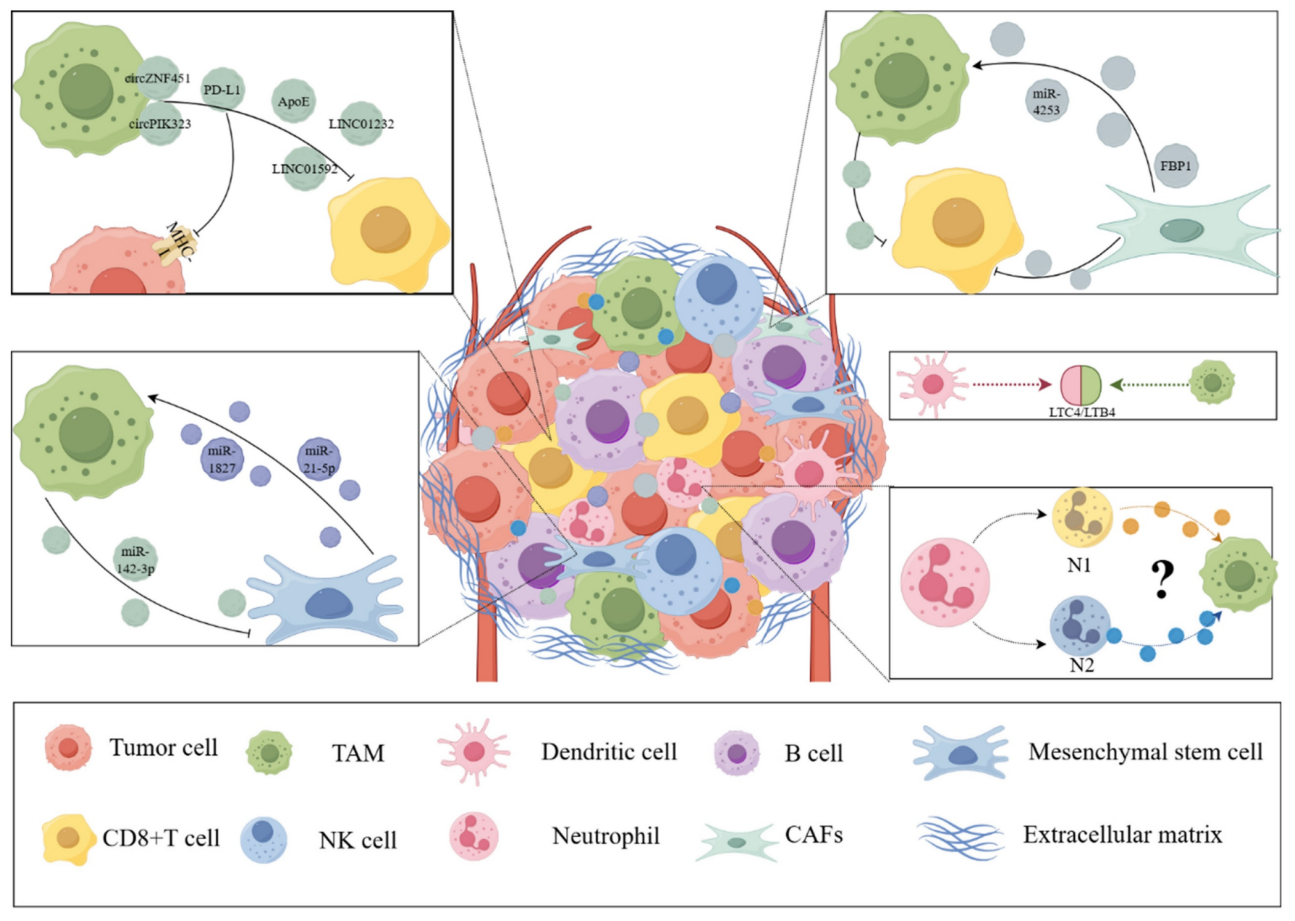
Exosomes in the TME are involved in the interaction between other cells and macrophages. Exosomes are widely involved in the exchange of information and material between the components of TME. CD8+ T cells, CAFs, neutrophils, and mesenchymal stem cells are also involved in the interaction between tumor cells and macrophages. These exosomes and their contents can provide an overall understanding of the crosstalk between the components of TME. (The Figure was drawn with the help of www.figdraw.com).

In addition to tumor cells, the TME comprises a complex environment composed of extracellular matrix, blood vessels, immune cells, and various signaling molecules and cytokines. These components all interact with macrophages in a complex manner, influencing the occurrence and development of tumors (*123*). In TME, cell interactions occur through direct contact and paracrine forms. Adhesion molecules, including integrins, cadherins, selectins, *etc,* mediate contact-dependent cell interactions. Paracrine signaling promotes intercellular communication by inducing the release of cytokines, chemokines, growth factors, and proteolytic enzymes from various cell types due to intrinsic tumor characteristics and cellular stress (*124*). Among them, EVs are another paracrine mechanism that can alter the TME. Exosomes derived from different components play a crucial role in this process, influencing tumor growth, metastasis, immune escape, angiogenesis, *etc.* (*125*). The following section summarizes and organizes the interactions between exosomes and other components of the TME, such as other immune cells and fibroblasts, with macrophages. This section will provide researchers a deeper understanding of the crucial role of macrophages in the TME and lay the foundation for exosome-based immunotherapy targeting macrophages (Figure 3).

### 3.1 Exosomes Participate in the Interaction between CD8+ T Cells and Tumor-associated Macrophages

In the TME, the interplay between T cells and macrophages is critical in influencing tumor development. These two cell types are essential for sustaining immune balance and assessing the functionality of other immune cells. Metabolic alterations in macrophages and T cells substantially impact their pro-tumor or anti-tumor activities by modulating signaling pathways and epigenetic modifications, making them potential targets for cancer immunotherapy (*126, 127*). Based on co-stimulatory factors, T cells can be classified into CD4+ T and CD8+ T cells. CD4+ T cells are activated by the complex formed by antigen peptides and class II molecules, becoming helper T cells.

Whereas CD8+ T cells are activated by the complex of antigen peptides and class I molecules, becoming cytotoxic T cells (CTLs), which can directly kill pathogen-infected and tumor cells. A key challenge in tumor immunotherapy is enhancing the recognition and killing functions of CD8+ T cells and overcoming the immune escape strategies of tumors (*128*). In HCC, Golgi membrane protein 1 (GOLM1) facilitates the deubiquitination of PD-L1, increasing the expression of PD-L1 on cell surfaces. Moreover, GOLM1 enhances the incorporation of PD-L1 into exosomes by inhibiting Rab27b in the trans-Golgi network, which increases exosomal PD-L1 levels. The PD-L1 contained within exosomes released from HCC cells boosts PD-L1 expression on TAMs, thus suppressing the activity of CD8+ T cells (*129*). Liu *et al.* also found that ERS-induced exosomes derived from HCC cells contain high levels of miR-23a-3p, which upregulates the expression of PD-L1 in macrophages by activating the PTEN/AKT pathway, thus reducing the ratio of CD8+ T cells and promoting T cell apoptosis (*38*). Wei *et al.* discovered that prostate cancer cells undergoing ERS have increased exosomal PD-L1 levels and activated PI3K/AKT pathway, which promote M2 polarization of macrophages and establish an immunosuppressive environment (*40*). Zhong *et al.* showed that PD-L1 expression in TAMs plays a key role in melanoma patients' response to PD-1 blockade immunotherapy. Furthermore, macrophage inhibition significantly improved the efficacy of PD-1/PD-L1 blockade in a mouse model, which was associated with increased intratumoral recruitment and enhanced cytotoxic CD8+ T cell functions. These findings suggest that therapies targeting macrophages can act synergistically with PD-1/PD-L1 blockade. Increased PD-L1 release from TAM-derived exosomes suppressed CD8+ T cell proliferation and function. Mechanistically, this inhibition is associated with Akt-mediated phosphorylation of MADD and elevated Rab27 activity. This finding indicates the potential clinical benefit of targeting and disrupting the exosome secretion pathway in TAMs to improve the efficacy of anti-PD-1 therapy (*130*). However, in OC, TAM-derived exosomes carrying PD-L1 can upregulate CPT1A in CD8+ T cells through the transcription factor PPARα, enhancing fatty acid oxidation and ROS production. These alterations promote T cell apoptosis and upregulate exhaustion markers LAG3 and TIM-3, thus inducing tumor peritoneal metastasis *via* immune imbalance (*131*). Compared with the above two mechanisms, the phosphorylation state of MADD and CPT1A is crucial for the interaction between macrophages and CD8+ T cells, as they induce mitochondrial ROS accumulation and epigenetic remodeling under TME exposure (such as low glucose and high lipid stress). The current clinical treatment includes PD-1/PD-L1 inhibitor monotherapy for melanoma, NSCLC, renal cell carcinoma, *etc*.; however, this treatment is limited by the response rate. Therefore, PD-1/PD-L1 inhibitor monotherapy is often combined with chemotherapy, targeted therapy, or double anti-PD-1 and CTLA-4 double immune checkpoint blockade. Based on this, TAM and exosome mechanisms should be comprehensively studied to improve the current treatment strategy (*132*). For example, incorporating the artificial intelligence prediction model to construct the interaction network between TAMs and CD8+T cells through single-cell sequencing data will provide a better understanding of the multi-directional regulatory role of PD-1/PD-L1 pathway in the interaction between macrophages and CD8+T cells and the role of exosomes in it, allowing the development of new drugs and combination strategies. It will guide personalized combination therapy, which would evolve tumor immunotherapy from "extensive activation" to "targeted remodeling".

In addition to the PD1/PD-L1 pathway, exosomes modulate the interactions between macrophages, T cells, and tumor cells within the TME *via* alternative regulatory mechanisms. Exosomes released by TAMs carry LINC01232, which contributes to tumor immune evasion. LINC01232 binds directly to E2F2, facilitating its nuclear translocation and enhancing the transcription of NBR1. This increases the binding of NBR1 to ubiquitinated MHC-I proteins through its ubiquitin-binding domain, increasing degradation of MHC-I in autolysosomes and reducing MHC-I surface expression on tumor cells. Therefore, this process diminishes CD8+ T cell-mediated antitumor immunity (*133*). Another study revealed that LINC01592, derived from exosomes, has a comparable regulatory impact. LINC01592 from M2 MDEs directly interacts with E2F6 in tumor cells, facilitating its nuclear translocation and boosting the transcription of NBR1. NBR1 utilizes its ubiquitin domain and strengthens the association between ubiquitinated proteins and MHC-I, resulting in increased degradation of MHC-I within autolysosomes and diminished surface expression of MHC-I on cancer cells. This promotes an effect analogous to the previously described mechanism, allowing for evasion of immune attacks by CD8+ T cells (*134*). M2 MDEs can transport ApoE, which interferes with the ATPase activity of binding immunoglobulin protein (BiP), which reduces MHC-I expression on tumor cells and inhibits the ability of CD8+ T cells to exert cytotoxic effects on these cells. As a result, tumor cells develop resistance to immune checkpoint blockade therapy. Whereas tumor cells' sensitivity to immune checkpoint inhibitors can be enhanced using the ApoE ligand EZ-482, which boosts the ATPase function of BiP. This restoration of BiP's function re-establishes tumor immunogenicity and improves the effectiveness of immunotherapy (*135*). This finding paves the way for future researchers to comprehensively investigate the potential of M2 MDEs loaded with EZ-482 in improving the effectiveness of immunotherapy and to devise more refined immunotherapy approaches. In LUAD, exosomal circZNF451 can induce an anti-inflammatory phenotype in macrophages and reduce cytotoxic CD8+ T cells by activating the ELF4-IRF4 pathway *via* TRIM56-mediated FXR1 degradation, altering the tumor immune microenvironment. Future studies could leverage M2 MDEs containing EZ-482 as a novel strategy to enhance immunotherapy outcomes (*85*). It has been reported that radiotherapy can promote the release of circPIK3R3 in melanoma exosomes, which, when absorbed by macrophages, promotes the secretion of type I interferon (I-IFN) and M1 polarization through the miR-872-3p/IRF7 axis. The secreted I-IFN activates the JAK/STAT signaling pathway in CD8+ T cells, promoting the secretion of IFN-γ and GZMB, and enhancing the anti-tumor immune response of CD8+ T cells (*136*). Therefore, in exosome-related immunotherapy protocols, inhibiting the exosome-related regulatory factors of macrophages affects MHC-I expression on the tumor cell membrane, improving the therapeutic effect of CD8+ T cell infusion, which presents a highly attractive prospect. The research and clinical translation in this domain can be further explored from the perspective of the interaction between macrophages and CD8+ T cells, providing novel strategies for real-world clinical treatment protocols.

### 3.2 Exosomes Participate in the Interaction between Fibroblasts and Tumor-associated Macrophages

The TME facilitates the growth and proliferation of tumor cells *via* multiple mechanisms. Tumor-associated fibroblasts (CAFs) and the extracellular matrix have been found to modulate these mechanisms. CAFs are one of the most abundant types of tumor stromal cells, which have significant functions in various malignant phenotypes (*137*). CAFs can stimulate the proliferation and invasion of tumor cells by secreting growth factors, chemokines, and EVs. This is consistent with the fact that macrophages and T cells in immune cells can also release pro-inflammatory cytokines to enhance tumor cells' survival ability, and there is a complex mutual regulatory effect. Ultimately, TME promotes tumor progression (*138, 139*). Exosomes, as important intercellular communication mediators, play a significant role in this process. Understanding the TME by considering stromal cells, macrophages, and tumor cells as soil, seed, and fertilizer helps researchers explore the TME's structure in-depth for developing more effective immunotherapy approaches.

Numerous studies have disclosed the interplay between macrophages and fibroblasts in the TME. For example, macrophages and fibroblasts both expressing SPP1+ exhibit complementary ligand-receptor pairs and might mutually affect their gene expression programs (*140, 141*); thus influencing tumor progression. Raymant *et al.* ascertained that the generation of pro-metastatic and immunomodulatory myofibroblasts (myMAFs) profoundly depends on macrophages. Mechanistically, myMAFs are driven by macrophage-derived progranulin and leukemia inhibitory factor secreted by cancer cells and are induced through a STAT3-dependent mechanism in an interactive manner. The secreted osteopontin promotes an immunosuppressive macrophage phenotype, suppressing cytotoxic T cell function. STAT3 inhibition can restore the anti-tumor immune response and reduce tumor metastasis (*142*). EVs serve as a crucial pathway for intercellular communication and have significant functions. A study showed that CAFs can transform primary human monocytes into immunosuppressive myeloid-derived suppressor cells (MDSCs) within a three-dimensional co-culture system. Inhibiting the generation of EVs in these CAF-induced MDSCs can counteract their suppression of T cell proliferation. Moreover, EVs extracted from CAF-induced MDSCs directly impair T cell functions. Furthermore, it has been shown that fructose-1,6-bisphosphatase 1 (FBP1) is abundantly present in the EVs derived from CAF-induced MDSCs. Pharmacologically inhibiting FBP1 can partially restore the immunosuppressive characteristics of MDSCs (*143*). In gastric cancer, CAFs secrete EVs that suppress M1 polarization and enhance M2 polarization of macrophages. Molecular studies have revealed increased expression of miR-4253 in CAF-derived EVs. Inhibiting miR-4253 can counteract the EV-induced changes in macrophage polarization. Further, IL6R has been identified as a downstream target of miR-4253, and exposing macrophages to EVs containing miR-4253 promotes gastric cancer cells' proliferation. Therefore, EVs carrying miR-4253 may represent a potential therapeutic target for gastric cancer treatment (*144*). Jiang *et al.* highlighted that in TME, exosomes originating from macrophages can facilitate metabolic interactions between fibroblasts and macrophages, influencing the tumor-killing efficacy of CD8+ T cells. Nicotinamide (NAM) and 1-methyl nicotinamide (MNAM) are two metabolites with contrasting effects in this environment. In clinical samples, elevated NAM levels have been observed to correlate with a favorable prognosis, whereas increased MNAM levels suggest a poorer outcome. The enzymes NNMT and NAMPT are rate-limiting in NAM metabolism and indicate opposing prognostic implications. Macrophages predominantly produce NAMPT in gastric cancer, while fibroblasts are the main source of NNMT. NAMPT from macrophages can suppress NNMT expression in CAFs *via* EVs. Researchers exposed CAFs to macrophage-derived EVs and co-cultured them with CD8+ T cells. This treatment significantly diminished the cytotoxic activity of CD8+ T cells; however, increasing the concentration of EVs restored T cell function. The underlying mechanism involves SIRT1 and NOTCH signaling pathways (*145*). The metabolic crosstalk between fibroblasts and macrophages in the TME mediated by EVs can be targeted for the immunotherapy of gastric cancer, and using EVs as a therapeutic approach to interrupt some intercellular communications can enhance the efficacy of immunotherapy.

### 3.3 Exosomes Participate in the Interaction between Other Cells and Tumor-associated Macrophages in the Tumor Microenvironment

Dendritic cells, like macrophages, can act as antigen-presenting cells and play a significant role in innate and adaptive immune responses (*146*). Dendritic cells can initiate the cancer/immune cycle and activate T cells to combat and eliminate tumor cells. Therapeutic methods targeting the immune response of dendritic cells to tumors have great potential. Several studies have investigated the role of exosomes in the communication between dendritic cells and tumor cells, as well as other immune cells. They proposed novel immunotherapy strategies based on exosomes, such as exosomes carrying the basic immune-stimulating functions of dendritic cells and influencing the antigen presentation of T cells (*147*). A review systematically summarized the significant regulatory effects of exosomes derived from dendritic cells and those derived from tumor cells, as well as the interaction between these two cellular components. Engineering strategies also hold great promise for enhancing the therapeutic efficacy of the immune therapy (*148, 149*). A comprehensive understanding of how the tumor immune microenvironment regulates tumor occurrence and development is crucial. As previously discussed, exosomes play a vital role in facilitating information exchange between macrophages and tumor cells, dendritic cells and tumor cells, as well as dendritic cells and macrophages. The contents of exosomes from dendritic cells are similar to those from macrophages. For example, leukotrienes (LTs), potent pro-inflammatory lipid mediators, can be synthesized by enzymes within exosomes released by both macrophages and dendritic cells. Macrophages primarily produce LTB4, whereas dendritic cells predominantly generate LTC4 (*150*). Since exosomes are crucially involved in the interaction between macrophages and tumor cells, it is necessary to incorporate dendritic cells and their derived exosomes into the research. Employing dendritic cell-derived exosomes to enhance the original macrophage-related immunotherapy is a promising strategy, as it influences the cytotoxicity of T cells at multiple levels and consequently improves the efficacy of immunotherapy.

Researchers have increasingly recognized the role of B cells in the tumor immune microenvironment. In addition to cooperating with T cells in immunity, B cells employ fundamentally distinctive mechanisms to discriminate self from non-self and elicit immune responses in multiple organs like the liver, kidneys, and lungs. They can influence the polarization of TAMs, constituting an essential aspect in tumor surveillance and control (*151*). In the TME, B cell metabolites and the small molecule substances they secrete can influence the anti-tumor effect of macrophages. Moreover, they can interact with other cellular components by secreting EVs (*152, 153*). For instance, in the subcapsular sinus of lymph nodes, B-cell-derived exosomes can be captured by CD169 macrophages and then penetrate deep into the cortex. The density of CD169-positive macrophages in lymph sinusoids is closely related to the density of infiltrating T or NK cells in tumor tissues, which is of great significance in the anti-tumor immune response of tumor patients (*154, 155*). Further in-depth studies on the role of B-cell-derived exosomes in the intricate TME environment, the interaction of immune cells such as macrophages and T cells, and the verification of their functions are required for developing novel immunotherapy strategies.

Neutrophils are crucially involved in the initiation and progression of cancer. They are analogous to TAMs and exhibit two distinct phenotypes (N1 and N2). Exosomes also mediate the interactions between neutrophils and tumor cells, influencing tumor progression (*156*). The distinct polarization states of neutrophils produce functionally divergent exosomes. Exosomes derived from N1 neutrophils are enriched in inflammatory biomolecules, whereas N2 neutrophil-derived exosomes contain regulatory biomolecules. N1 neutrophil-derived exosomes promote macrophage activation and T cell proliferation, while N2 neutrophil-derived exosomes induce M2 polarization of macrophages (*157*). However, the role of neutrophil-derived exosomes and their interactions with macrophages in cancer therapy remains undetermined, primarily due to the short half-life and low yield of neutrophils. Despite these challenges, the similarities between neutrophil-derived exosomes and TAMs, along with the unique ability of neutrophil-derived exosomes to cross the bone marrow barrier, indicate their significant potential for engineered strategies in cancer treatment (*158, 159*).

Mesenchymal stem cells (MSCs) are multipotent stromal cells commonly found in TME and play a crucial role in shaping the TME and promoting tumor progression. Tumors can exploit exosomes to reprogram MSCs, converting them into tumor-associated MSCs (*160*), which in turn influence fibroblasts, endothelial cells, and immune cells within the TME, indirectly improving their pro-tumor functions (*161*). Under the influence of tumor-derived exosomes, MSCs can be directly reprogrammed into M2-polarized macrophages, which further contribute to the tumor (*162*). In lung cancer, under hypoxic conditions, the overexpressed miR-21-5p delivered by exosomes from pre-human bone marrow mesenchymal stem cells (BMMSCs) can suppress pro-apoptotic genes (such as PTEN, PDCD4, and RECK) and induce M2 polarization of macrophages, exerting pro-proliferative and pro-metastatic effects (*163*). The miR-1827 derived from exosomes of MSCs can modulate succinate receptor 1 (SUCNR1). SUCNR1 downregulation can inhibit M2 macrophage polarization and suppress CRC cells' proliferation, migration, and invasion properties. Moreover, *in in vivo* experiments, it blocks liver metastasis of CRC (*164*). Radiotherapy is a significant modality in cancer treatment; however, it inevitably inflicts damage upon the surrounding normal tissues. Bone tissue is one of the common targets of radiation injury. BMMSCs are sensitive to radiation, and their functional disorders play a pivotal role in radiation-induced bone injury. The M2 MDEs inhibit the fibrotic differentiation of irradiated BMMSCs *via* the miR-142-3p/TGF-β1 axis and restore their osteogenic differentiation balance, mitigating radiation-induced bone injury (*165*).

## 4. Engineering Strategies and Biomaterials Combined with Exosome Therapy are the Therapeutic Frontiers in This Field

**Figure 4:**
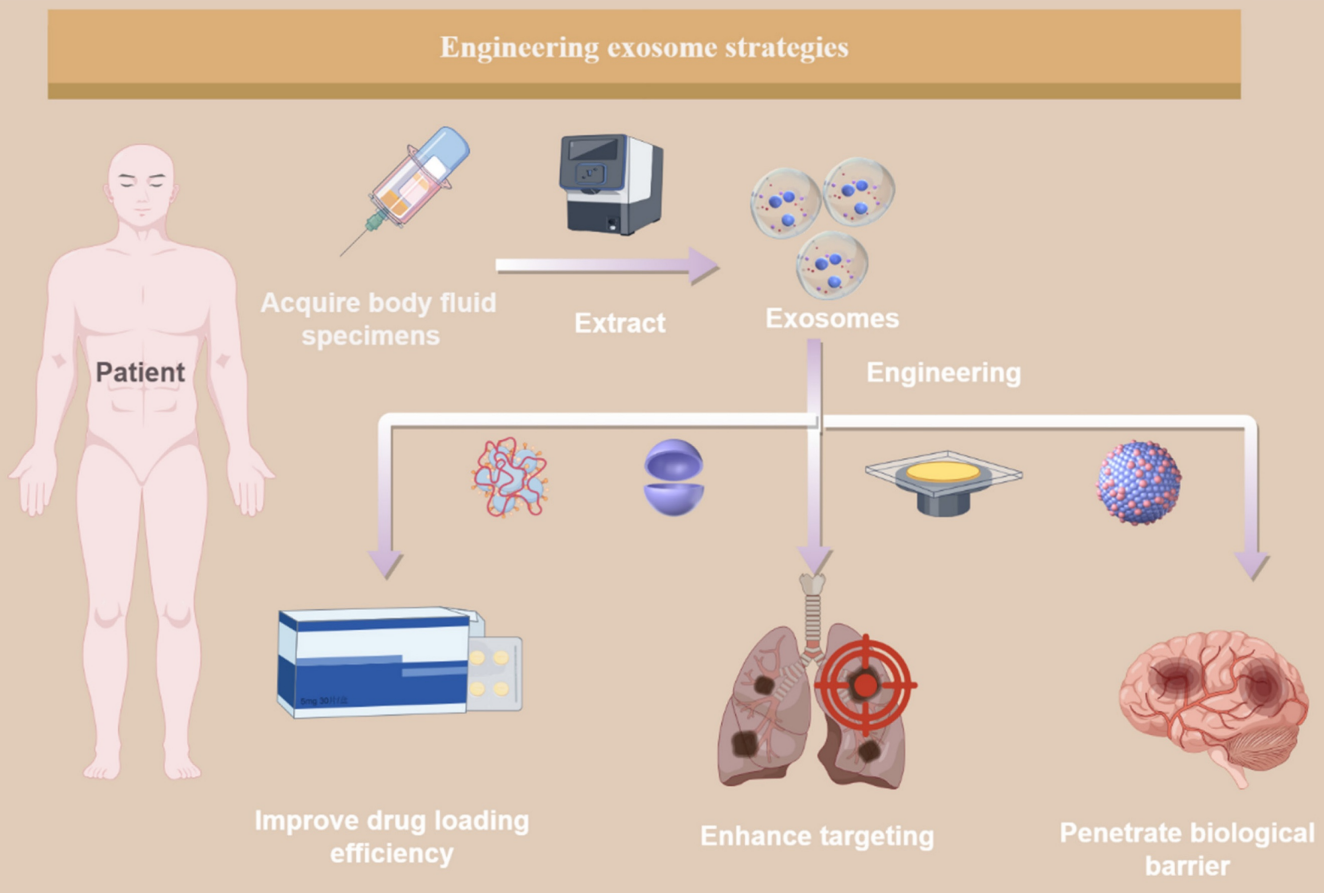
The application of engineered exosome strategies in TME research. Exosomes are extracted from human blood, saliva, urine, *etc.,* and modified by engineering materials methods to effectively improve the drug loading rate, targeting, and biofilm penetration of exosome therapy, and improve the effect of tumor immunotherapy. (The Figure was drawn with the help of www.figdraw.com).

Exosomes modulate the communication between macrophages and tumor cells, and their functions are determined by the specific molecules they carry. Research in tumor therapy has introduced innovative concepts and approaches for cancer treatment. A deeper understanding of how exosomes operate within the TME can help develop more effective cancer therapies in the future. According to data from the ClinicalTrials.gov website (https://www.clinicaltrials.gov), there are over 200 ongoing clinical trials related to exosomes. As an essential medium for information and material exchange between different components of the TME and macrophages, exosomes can be employed to develop diagnostic and therapeutic strategies based on the TME. However, challenges such as exosome stability, content loading, targeted delivery, and production and purification processes limit their widespread application in clinical immunotherapy regimens. Despite these obstacles, advancements in exosome engineering have shown promising results in increasing the therapeutic efficacy of exosomes. The following section will integrate current material on engineering techniques to improve tumor diagnosis and treatment using exosomes and provide guidance for the clinical translation of future therapeutic strategies (Figure 4).

The engineering of macrophage exosomes has developed into a multi-dimensional technical field. Based on the differences in targeting specificity, modification method, and delivery efficiency, different engineering materials science strategies can markedly improve the individualization and effectiveness of tumor immunotherapy. First, specific receptor-ligand interactions can be used for precise targeting and improving the effect of immunotherapy. The interleukin-4 receptor (IL4R) expression was higher in M2 TAMs than in anti-tumor M1 macrophages. Gunassekaran *et al.* constructed a vector using the IL4R-binding peptide IL4RPep-1 to target this specific receptor and successfully developed IL4R-Exo. This modified exosome vector showed a strong efficacy in reducing the expression of M2 macrophage target genes. Furthermore, whole-body fluorescence imaging showed that IL4R-Exo could be significantly aggregated in tumor tissues, and could inhibit tumor growth, reduce M2 cytokines and immunosuppressive cells, and increase M1 cytokines and immune-stimulating cells. Reprogramming TAMs into M1-type macrophages and improving anti-tumor immune response can effectively inhibit tumor progression, which provides strong support for tumor immunotherapy (*166*). As aforementioned, M2 phenotype regulation is mainly dependent on key transcription factors such as STAT6. Exosome engineering strategies can be employed to improve the specificity of targeting these factors in TAMs. Kamerkar *et al*. employed exosomes carrying antisense oligonucleotides (ASOs) targeting STAT6 (exoASO-STAT6) to silence STAT6 expression in TAMs. In both colorectal and HCC models, exoASO-STAT6 treatment inhibited tumor growth by > 90%, induced an increase in the M1 macrophage marker nitric oxide synthase 2, and reshaped TME to generate CD8+ T cell-mediated adaptive immune responses (*167*). Wang *et al.* developed chimeric exosomes produced by macrophage-tumor hybrid cells (aMT-exos) and indicated the ability to target tumors and lymph nodes. These exosomes stimulated T cells through the antigen presentation pathway and exosome interaction, effectively inhibiting the growth and metastasis of various mouse tumor models. The combination of PD1 and PD1 can significantly improve the therapeutic effect on tumors, and provide a new way for tumor immunotherapy (*168*). Studies have reported a polylactic acid-co-glycolic acid (PLGA) nanoparticle system loaded with MDEs, which conjugated c-Met peptides abundantly expressed in triple-negative breast cancer cells, to the surface of exosomes. These nanoparticle-coated exosomes significantly increased the cellular uptake efficiency and the antitumor effect of doxorubicin (*169*). Moreover, researchers have also developed and modified a paclitaxel (PTX)-loaded exosome incorporating an aminoethylbenzoyl-amide-polyethylene glycol (AA-PEG) carrier moiety to target sigma receptors overexpressed in lung cancer cells. When administered systemically, PTX-loaded exosomes and AA-PEG-loaded exosomes (AA-PEG-ExopTX) show strong targeting ability, accumulate in cancer cells, and improve the therapeutic effect (*170*).

**Figure 5:**
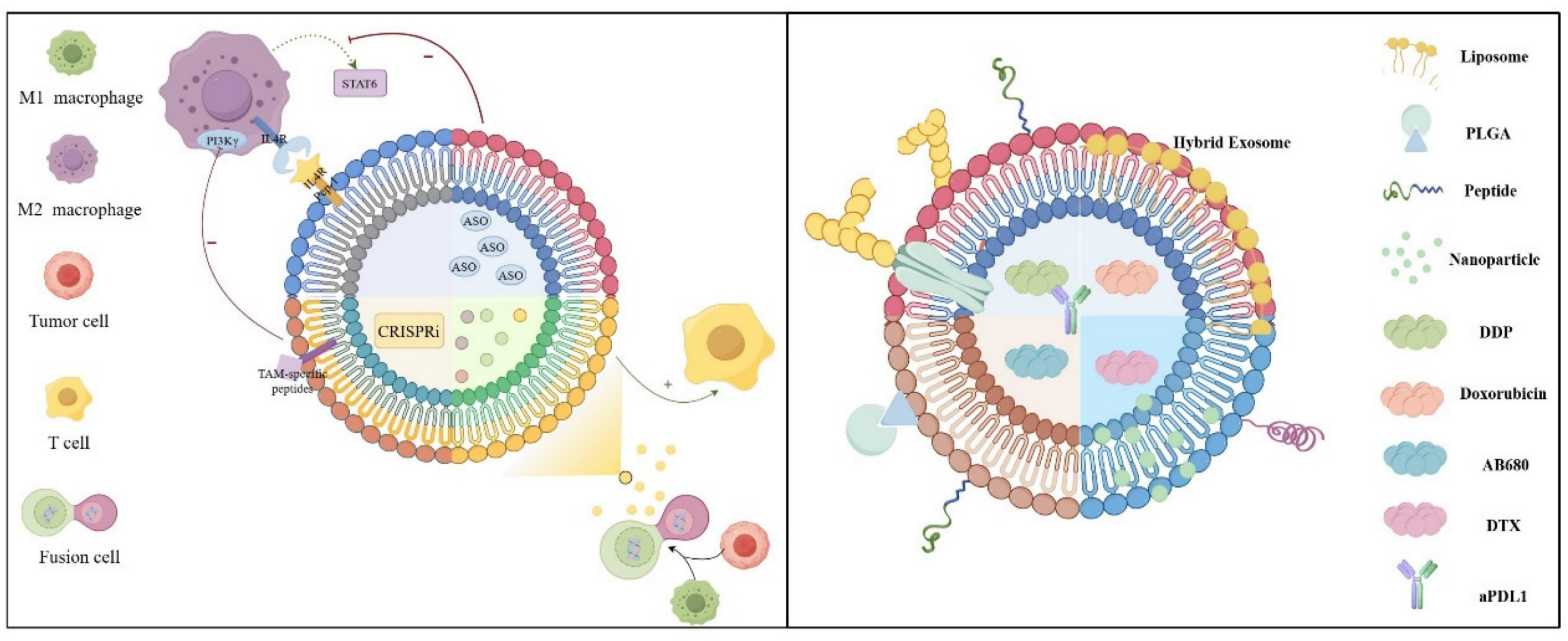
Schematic diagram of engineering materials science methods for modifying exosomes. In specific engineering strategies, cell surface receptors can be loaded on the membrane structure of exosomes, or gene editing can be used to modify exosomes to improve their targeting. Furthermore, chemotherapy and immunotherapy drugs can be loaded into exosomes to combine the advantages of nanoparticles to improve the therapeutic effect of drugs. (The Figure was drawn with the help of www.figdraw.com).

Gene editing and nucleic acid delivery have been extensively studied for many years. Researchers can achieve long-lasting phenotypic reprogramming through engineered exosome-regulated gene pathways and completely change immunotherapy strategies. A study developed exosomes containing clustered regularly interspaced short CRISPris for internal modification and TAM-specific peptides for external modification on the exosome membrane. These internal and external modified exosomes (called IEEE or I3E) could selectively target tumor tissues and M2-TAMs. They effectively inhibit PPI3Kγ expression and promote TAM polarization to the M1 phenotype *in vivo* and the laboratory. The resulting M1-polarized macrophages enhanced "hot" tumor immunity, which resulted in increased T-lymphocyte infiltration, reduced numbers of myeloid-derived suppressor cells, and significant suppression of tumor growth (*171*). Choo *et al.* used nanovesicles that mimic M1 macrophage exosomes (M1NVs) to reprogram M2 TAMs into M1 macrophages, which secrete proinflammatory cytokines and stimulate antitumor immune responses. M1NVs treatment effectively converted M2-type macrophages to M1 phenotype* in vitro* and *in vivo*. Intravenous injection of M1NVs in tumor-bearing mice inhibited tumor growth. Furthermore, the combination of M1NVs and PD-L1 inhibitors significantly reduced tumor volume compared with either treatment alone (*172*). In these engineered strategies to modify macrophage phenotypes and cell-to-cell interactions through gene editing or nanotechnology, researchers should carefully evaluate off-target effects to avoid unpredictable adverse events and improve therapeutic efficacy. Zhen *et al.* developed a novel hybrid nanovesular (P-I@M1E/AALs) targeting the cancer-promoting properties of TAMs in the hypoxic and immunosuppressive microenvironment of tumors such as triple-negative breast cancer. By integrating M1-type macrophage exosomes with targeted aptamer-modified liposomes loaded with perfluorobutylamine (PFTBA) and photosensitizer IR780, this platform achieves multiple synergistic therapies. Furthermore, it alleviated tumor hypoxia, reprogrammed TAMs to anti-tumor phenotypes, improved photodynamic therapy, and activated T lymphocyte infiltration. TME can significantly inhibit tumor growth and prolong the survival time of mice, indicating that it can be employed as a multimodal treatment strategy for overcoming hypoxia and immunosuppressive TME (*173*) (Figure 5).

By employing engineered exosome therapy and traditional cancer treatment, the multifunctional combined therapy platform can integrate chemotherapy drugs and immunomodulatory functions to improve the therapeutic effects of the chemotherapy-immune synergy system. For example, one study developed a novel anti-tumor strategy by encapsulating docetaxel in M1-derived exosomes (DTX-M1-Exo). This engineered exosome can convert undifferentiated M0 macrophages into M1 macrophages with antitumor effects, and this transformation state remains stable when exposed to M2-inducing IL-4, preventing the transformation to tumor-progression-promoting M2 macrophages. This dual mechanism of action enables DTX-M1-Exo to synergize the advantages of chemotherapy and immunotherapy as a promising tumor treatment (*174*). Using MDEs-like nanoveses (EMVs), researchers have established a novel nanodelivery platform to simultaneously deliver a CD73 inhibitor (AB680) and an anti-PDL1 (aPDL1) monoclonal antibody. This complex (AB680@EMVs-aPDL1) is stable and biosafe. It showed significant tumor targeting in bladder cancer mice. In terms of therapeutic effect, AB680 inhibited adenosine production, and combined with aPDL1 treatment, significantly activated and promoted the infiltration of cytotoxic T lymphocytes into the tumor, effectively inhibited tumor growth, and prolonged the survival time of the mice (*175*). Furthermore, investigators designed a new method for using MDEs to deliver paclitaxel (PTX, exoPTX) and optimized the drug delivery and sustained release of paclitaxel by sonication. *In vitro* experiments showed that exoPTX increased the cytotoxicity of MDCKMDR1-resistant cells by > 50-fold. exoPTX can effectively target tumor cells and significantly inhibit tumor growth in a Lewis lung metastatic cancer mouse model. This technology holds promise as a novel chemotherapeutic delivery platform for the treatment of drug-resistant cancers (*176*). These multi-functional combined platform systems provide promising strategies for increasing the effect of chemotherapy alone. In the study by Rayamajhi and colleagues, small vesicles isolated from mouse macrophages were combined with synthetic liposomes to form hybrid vesicles < 200 nm, replicating the size of native exosomes. These engineered vesicles, termed hybrid exosomes (HE), were loaded with water-soluble doxorubicin. HE has increased toxicity to cancer cells and promotes pH-sensitive drug release in an acidic environment, increasing drug delivery to acidic TME (*177*). Using electroporation, the investigators incorporated cisplatin into M1 MDEs (DDP-M1-Exos) and confirmed that it was efficiently internalized by lung cancer cells. *In vitro* studies showed that DDP-M1-Exos was significantly superior to free cisplatin (DDP) in inducing lung cancer cells' apoptosis by up-regulating apoptosis-related proteins, including Bax and Caspase-3. Moreover, *in vivo* experiments revealed that M1-Exos had anti-tumor properties, and DDP-M1-Exos loaded with cisplatin significantly increased their anti-lung cancer effect. M1 MDEs can be used as an efficient delivery system for cisplatin to improve the therapeutic effect on lung cancer (*178*).

Exosomes can also solve the problem of tumor drug resistance and the limited drug treatment effect. To overcome gemcitabine (GEM) resistance in pancreatic cancer, researchers have developed a drug delivery system using M1 MDEs (M1Exo). When added to M1Exo, deferasirox (DFX) reduces the expression of the ribonucleotide reductase regulatory subunit through iron ions within the chelate, improving the chemotherapeutic effect of GEM. This M1exo-based nanodrug, loaded with GEM and DFX, indicated significant therapeutic effects in PANC-1/GEM resistant cells and 3D tumor sphere models (*179*).

For nervous system tumors, the blood-brain barrier (BBB) is highly selective and restricts various drugs from entering the brain, thus making the treatment of brain tumors extremely challenging. Researchers have developed a novel nanoplatform, CSI@Ex-A, which utilizes macrophage exosomes to deliver silica nanoparticles loaded with catalase (CAT) and the photosensitizer indocyanine green (ICG) to enhance BBB penetration and tumor targeting. CAT catalyzes oxygen generation to alleviate tumor hypoxia and improve the efficacy of sonodynamic therapy (SDT) (*180*). In diffuse endogenous pontine glioma (DIPG), p53-induced protein phosphatase 1 (PPM1D) gene mutation promotes tumor cell proliferation. Knockdown of PPM1D expression in PPM1D mutant DIPG cells significantly inhibited their proliferation ability. Although Panobinostat exhibits potent cytotoxicity against DIPG cells, its clinical use is limited by systemic toxicity and poor BBB penetration. To address these challenges, a nanodrug delivery system was developed that utilizes engineered macrophage exosomes and delivers Panobinostat and PPM1D-specific small interfering RNA to provide targeted therapy for PPM1D-mutated DIPG (*181*).

## 5. Summary and Outlook

This review discussed the polarization of M1 and M2 macrophages and the role of exosomes in shaping TME for tumor progression. Furthermore, the effect of tumor-derived exosomes on macrophage polarization was studied, and the mechanisms under conditions of hypoxia and ERS were discussed. In various types of cancer, exosomes released by different tumor cells can promote M1 (anti-tumor) or M2 (pro-tumor) macrophage polarization through multiple pathways involving miRNAs, lncRNAs, and proteins. TME should be considered when studying the interaction between tumor cells and macrophages. This includes exploring the interaction of exosomes from M1 and M2 macrophages with tumor cells and other immune cells, such as CD8+ T cells, fibroblasts, dendritic cells, and B cells. To understand these complex interactions, the heterogeneity of exosomes derived from different cellular sources should be considered. A comprehensive understanding of immune cells, stromal cells, tumor cells, and their exosomes can provide a basis for the development of immunotherapy strategies. Exosomes can also be used as non-invasive biomarkers for early diagnosis of cancer and monitoring disease progression. For example, Extracellular Small molecule Chemical Occupancy and Protein Expression monitoring (ExoSCOPE) uses bioorthogonal probe amplification and matches the spatial pattern of molecular reactions in plasma nanoring resonators for *in situ* analysis of EV pharmacokinetics. Furthermore, it can measure drug occupancy and protein composition changes in EV molecular subgroups and monitor the effects of targeted cell therapies. It has the advantage of employing a small amount of sample, accurately classifying disease status, and rapidly differentiating targeted therapy outcomes within 24 hours of treatment initiation (*182*). Several new technologies and studies have highlighted the advantages of exosomes in ultra-early diagnosis and dynamic monitoring.

The engineering approach can also increase the therapeutic potential of exosome therapy in cancer immunotherapy. The strategy of engineered exosomes is gradually moving from basic research to preclinical and clinical stages. The core breakthrough of its translational pathway is focused on the synergistic optimization of three major strategies: targeted modification technology, drug delivery system innovation, and dynamic regulation design. By precisely regulating the biological characteristics and functions of exosomes, these strategies can solve the problems of off-target effects of traditional therapies, low drug delivery efficiency, and adaptive resistance of TME. Through surface engineering of exosomes *via* gene editing or chemical conjugation technology, tumor-homing molecules (such as specific peptides or single-chain antibodies) can be displayed on the surface of the exosome membrane. These molecules can accurately recognize tumor cells or specific immune cell surface markers, enhance the accumulation ability of exosomes at the lesion site, and combine multivalent binding strategies. It can break through the complex physical barriers in the TME and significantly improve the precision of therapeutic load delivery. As natural nanocarriers, the drug loading strategy of exosomes is expanding from single drug loading to multimodal collaborative therapy. Efficient co-loading of small-molecule drugs, nucleic acids, and protein drugs can be achieved by physical (*e.g.,* electroporation, ultrasonic perturbation) or chemical (membrane fusion, hydrophobic interaction) methods. Considering the tumor heterogeneity and immunosuppressive microenvironment, the "multi-warhead" drug delivery system is designed to simultaneously target tumor cell proliferation, immune escape, and matrix remodeling. Similarly, by regulating the fluidity of the exosome membrane and the microenvironment of the inner cavity, the biodegradable drug can be stabilized, and its half-life *in vivo* can be prolonged. Intelligent responsive exosomes based on TME-specific signals (such as low pH, high ROS, or specific enzyme activity) can also overcome tumor adaptive drug resistance. Such exosomes can integrate environmentally sensitive molecular switches through modular design to release therapeutic loads at the lesion site on demand. For example, in tumor cells, acidic lysosomes stimulate the exosome membrane structure to undergo phase transition or degradation to release the encapsulated drug rapidly or to activate the functional domain of an immunomodulatory factor by digestion of a responsive linker arm in a TME overexpressing matrix metalloproteinases.

Based on the above conclusions, the following prospects were concluded for the key challenges and solutions of future research:

1. Exosomes derived from various cellular components are incorporated within the TME. Exosomes have significant heterogeneity, and depending on their cellular origin within the TME, their molecular composition can inhibit or promote immune responses. Therefore, drawing universal conclusions can be challenging, and incorporating different exosomes into studies may yield conflicting results. For example, exosomes from TAMs are enriched with miR-21 and miR-29-3p. When mimics of these miRNAs are transfected into CD4+ T cells, they induce an imbalance in Treg/Th17 cell populations *via* STAT3 signaling, leading to an immunosuppressive microenvironment at the tumor site, which facilitates tumor progression and metastasis (*183*). To address this challenge, single-exosome sequencing technology and spatial multi-omics integration analysis should be developed, combined with a CRISPR screening platform to identify the molecular signatures of specific exosome subsets and solve exosome functional heterogeneity.

2. In clinical settings, exosomes extracted from body fluids have greater complexity and significant uncertainties. Furthermore, the isolation and purification processes for exosomes currently lack stringent, clinically validated standards that ensure both safety and efficacy. Ensuring adequate clinical yields of exosomes for subsequent identification and therapeutic applications is challenging. Moreover, stable transportation and long-term preservation further complicate these issues, substantially reducing the economic viability and practicality of exosome-based therapies. Therefore, an "exosome control cloud platform" based on artificial intelligence should be established to integrate nanoflow and proteomics data and realize batch-to-batch stability monitoring for comprehensive research on exosomes and better clinical translation.

3. There is a lack of in-depth studies on the role of neutrophil-derived exosomes in TME because of the limited efficiency of exosome extraction or their half-life. Therefore, more research should be devoted to improving the extraction method of exosomes. In addition to the common methods, such as ultra-high speed centrifugation and ultrafiltration, many studies have employed novel strategies to solve the difficulties and challenges in this field. Zhang *et al.* presented a rapid magnetic separation method to isolate exosomes from neutrophils and engineer them with dual tumor targeting (biological and magnetic) (*159*). Researchers should refer to this research, as it demonstrates the role of exosomes in the anti-tumor activity of neutrophils, and also provides natural and biomimetic engineering strategies as new cancer therapeutic agents and drug delivery nanoplatforms.

4. The immunogenicity and potential toxicity of exosomes cannot be overlooked. To truly integrate exosomes into clinical therapies, it is imperative to address and mitigate their immunogenic and toxic effects on recipients during the preclinical research phase. In 2019, the FDA issued a Public Safety Notification on Exosome Products (https://www.fda.gov/vaccines-blood-biologics/safety-availability-biologics/public-safety-notification-exosome-products), highlighting concerns regarding their safety. MISEV2023 suggests that engineering modifications such as targeting peptides or antibody conjugation may change the immune characteristics of EVs, and the immune safety of nucleic acid loading needs to be verified in functional studies. The assessment of EVs' immunogenicity and toxicity should avoid risks through accurate design, standardized operation, and transparent reporting. Therefore, it is necessary to further establish the EVs' toxicity database and develop high-throughput screening technologies to accelerate clinical translation (*184*). Moreover, for safe clinical translation of exosome therapy, it is crucial to develop organoid exosome interaction models to simulate the *in vivo* toxicity and build toxicity databases to predict the immunogenicity risk.

To address these challenges, section 4 proposes that engineered strategies have great potential for clinical translation in exosome-based therapeutics. Although much work remains to be done, engineered exosomes have already made substantial progress in addressing key issues, including endogenous and exogenous loading methods, enabling efficient encapsulation of cargo. Moreover, they facilitate the monitoring and evaluation of key parameters such as drug loading, cellular uptake, in vivo distribution, and pharmacokinetics, thus increasing the efficiency of exosomes as therapeutic agents.

As specialists in oncology, our team recognizes that to overcome current limitations and challenges, it is essential to understand the full clinical potential of exosomes, which will require continued interdisciplinary collaboration between bioengineers, pharmacologists, and clinicians. Future exosome research must break disciplinary barriers, combine artificial intelligence (AI) and machine learning (ML) tools to understand exosomes and their complex contents, and distinguish patient populations. Multidisciplinary data (clinical, genetic and molecular) are used to explore the key targets in the exosomes-immune cell interaction network driven by bioinformatics, build a "diagnosis-treatment-monitoring" integrated platform to realize the closed loop of clinical transformation, and establish a "risk level-adaptive approval" pathway to accelerate the marketing process of exosome therapeutic drugs. These innovations can increase the likelihood of successful treatment outcomes while minimizing potential adverse effects, thus improving the overall landscape of cancer treatment. In summary, exosome research is at a key inflection point from mechanism exploration to clinical transformation. Exosomes can reshape the landscape of precision cancer medicine in the future through engineering strategies to solve the delivery bottleneck, through multi-omics technology to unlock the diagnostic potential, and through intelligent platforms to optimize the treatment paradigm.

## Figures and Tables

**Table 1:** Tumor-derived exosomes facilitate the polarization of macrophages towards the M2 or M1 phenotype.

Cancer	Exosome contents	Mechanism	Reference
head and neck squamous cell carcinoma	miR-29a-3p	SOCS1/STAT6	56
	CMTM6	ERK1/2	57
	lncRNA UCA1	miR-134/PI3K/AKT	58
nasopharyngeal carcinoma	RNF126	PI3K/AKT	61
esophageal squamous cell carcinoma	miR-301a-3p	PI3K/AKT	62
gastric cancer	miR-4435-2HG	Jagged1/Notch, JAK1/STAT3	63
	miR-92a-3p	ERK	67
	miR-519a-3p	DUSP2/MAPK/ERK	66
pancreatic cancer	FGD5-AS1	STAT3/NF-B	68
	KRAS ^G12D^	AGER/STAT3	69
colorectal cancer	miR-106b	PI3K/AKT/mTOR	71
	miR-934	PI3K/AKT	72
	miR-25-3p, miR-130b-3p, miR-425-5p	PI3K/AKT	74
hepatocellular carcinoma	miR-200b-3p	JAK/STAT	78
	miR-21-5p	MAPK/ERK	79
	miR-146a-5p	STAT3	80
lung cancer	miR-19b-3p	PTPRD/STAT3	83
	circZNF451	ELF4-IRF4	85
renal cell carcinoma	LncRNA-ARSR	STAT3	90
	AP000439.2	STAT3/NF-κB	91
cervical cancer	TIE2	AKT/PAK1	94
	miR-423-3p	STAT3	95
ovarian cancer	miR-200b	KLF6	97
breast cancer	miR-138-5p	KDM6B	99
	miR-222	PTEN/AKT	100
glioma	miR-3591-3p	JAK2 / PI3K / AKT / mTOR, STAT3	103
hematologic tumor	GP130	STAT3	104
	circ001264	STAT3	105
head and neck squamous cell carcinoma	miR-9	PPARδ	45
oral squamous cell carcinoma	THBS1	P38, AKT, SAPK/JNK	46
breast cancer	PTPRO	STAT3 and STAT6↓	47
	PEDF	-	48
	miR-33	-	49
	miR-130, miR-33	-	51

**Table 2:** The mechanism of M2 macrophage-derived exosomes in influencing tumor progression.

Cancer	Exosome contents	Mechanism	Effects	Reference
gastric cancer	ApoE	PI3K/Akt	Promote the migration of tumor cells	112
esophageal carcinoma	AFAP1-AS1	miR-26a/ ATF2	Promote the migration, invasion and lung metastasis of tumor cells	113
cholangiocarcinoma	circ0020256	miR-432-5p/E2F3	Proliferation, migration and invasion	114
hepatocellular carcinoma	miR-660-5p	KLF3	Proliferation, migration and invasion	115
	miR-27a-3p	TXNIP	Enhance the stemness of tumor cells	116
	miR-23a-3p	PTEN/TJP1	Promote EMT and angiogenesis as well as increase vascular permeability	117
	MAPKAPK5-AS1	c-Myc	Proliferation	118
	lncMMPA	miR-548/ ALDH1A3	Proliferation	119
colorectal cancer	miR-21-5p, miR-155-5p	BRG1	Migration and invasion	8
	miR-155-5p	ZC3H12B	Promote the immune escape of tumors	120
pancreatic ductal adenocarcinoma	miR-155-5p, miR-221-5p	E2F2	Proliferation and angiogenesis	121
	miR-501-3p	TGF-β/ TGFBR3	Proliferation, migration and invasion	122
lung cancer	miR-942	FOXO1/β-catenin	Migration, invasion, and angiogenesis	185
	miR-1911-5p	CELF2/ZBTB4	Migration and invasion	186
	miR-3679-5p	NEDD4L/c-Myc	Chemotherapy resistance	187
meningioma	-	TGF-β	Proliferation, migration, invasion and angiogenesis	188
ovarian cancer	circTMCO3	miR-515-5p/ITGA8	Proliferation, migration and invasion	189
cervical cancer	LRRC75A-AS1	miR-429/SIX1/ STAT3/MMP-9	Proliferation, migration and invasion	190
osteosarcoma	PDE4C	COL11A2, COL9A1, COL9A3	Proliferation, migration and immunotherapeutic effects	191

## Abbreviations

TME: The tumor microenvironment
TAMs: tumor-associated macrophages
CRISPRi: palindromic repeats interference
ERS: endoplasmic reticulum stress
LPS: lipopolysaccharide
PD-L1: programmed death ligand 1
ILVs: multiple intraluminal vesicles
HIFs: Hypoxia-inducible factors
OXPHOS: oxidative phosphorylation
HCC: hepatocellular carcinoma
GRP78: glucose-regulated protein 78
ATF6: activating transcription factor 6
PERK: PKR-like endoplasmic reticulum kinase
IRE1α: inositol-requiring enzyme 1α
HNSCC: neck squamous cell carcinoma
OSCC: oral squamous cell carcinoma
IONs: Iron oxide nanoparticles
CSCs): Cancer stem cells
ESCC: esophageal squamous cell carcinoma
PDCD4: programmed cell death 4
xCT: cystine-glutamate
GOLM1: Golgi membrane protein 1
BiP: binding immunoglobulin protein
CAFs: tumor-associated fibroblasts
MDSCs: myeloid-derived suppressor cells
NAM: Nicotinamide
LTs: leukotrienes
MSCs: Mesenchymal stem cells
SUCNR1: succinate receptor 1
BMMSCs: Bone marrow mesenchymal stem cells
IL4R: nterleukin-4 receptor
ASOs: antisense oligonucleotides
aMT-exos: chimeric exosomes generated from macrophage-tumor hybrid cells
M1NVs: exosomes from M1 macrophages
HE: hybrid exosomes
TNBC: triple-negative breast cancer
PLGA: polylactic-co-glycolic acid
PTX: paclitaxel
AA-PEG: aminoethylbenzoyl amide-polyethylene glycol
aPDL1: anti-programmed cell death ligand 1
DDP: cisplatin
DFX: Deferasirox
DTX-M1-Exo: docetaxel within exosomes derived from M1-type macrophages
CAT: silica nanoparticles loaded with catalase
ICG: photosensitizer indocyanine green
SDT: sonodynamic therapy
DIPG: diffuse intrinsic pontine glioma
PPM1D: p53-induced protein phosphatase 1
BBB: blood-brain barrier
